# Liquid Chromatography-Tandem Mass Spectrometry for the Simultaneous Analysis of 353 Pesticides in the Edible Insect *Tenebrio molitor* Larvae (Mealworms)

**DOI:** 10.3390/molecules25245866

**Published:** 2020-12-11

**Authors:** Yongho Shin, Chang Jo Kim, Sujin Baek, Leesun Kim, Kyeong-Ae Son, Hee-Dong Lee, Danbi Kim, Jeong-Han Kim, Hyun Ho Noh

**Affiliations:** 1 Residual Agrochemical Assessment Division, National Institute of Agricultural Sciences, Rural Development Administration, Iseo-myeon, Wanju-gun 55365, Jeollabuk-do, Korea; yong6103@korea.kr (Y.S.); rlawkdwh1@gmail.com (C.J.K.); bsj920@gmail.com (S.B.); twosuns@korea.kr (L.K.); sky199@korea.kr (K.-A.S.); yihd@korea.kr (H.-D.L.); danbi6334@korea.kr (D.K.); 2 Pesticide Chemistry and Toxicology Laboratory, Department of Agricultural Biotechnology and Research Institute of Agriculture and Life Sciences, Seoul National University, Seoul 08826, Korea; kjh2404@snu.ac.kr

**Keywords:** pesticide, multiresidues, edible insects, mealworms, LC-MS/MS, QuEChERS, acetonitrile-hexane partitioning

## Abstract

*Tenebrio molitor* larvae (mealworm) is an edible insect and is considered a future food. Using liquid chromatography-tandem mass spectrometry (LC-MS/MS), a novel method for simultaneous analysis of 353 target analytes was developed and validated. Various sample preparation steps including “quick, easy, cheap, effective, rugged, and safe” (QuEChERS) extraction conditions, number of acetonitrile-hexane partitions, and dispersive-solid phase extraction (dSPE) sorbents were compared, and the optimal conditions were determined. In the established method, 5 g of homogenized mealworms was extracted with acetonitrile and treated with QuEChERS EN 15662 salts. The crude extract was subjected to three rounds of acetonitrile-hexane partitioning, and the acetonitrile layer was cleaned with C18 dSPE. The final solution was matrix-matched and injected into LC-MS/MS (2 μL). For target analytes, the limits of quantitation (LOQs) were ≤10 μg/kg, and the correlation coefficient (*r^2^*) of calibration was >0.990. In recovery tests, more than 90% of the pesticides showed an excellent recovery range (70–120%) with relative standard deviation (RSD) ≤20%. For more than 94% of pesticides, a negligible matrix effect (within ±20%) was observed. The analytical method was successfully applied and used for the detection of three urea pesticides in 4 of 11 mealworm samples.

## 1. Introduction

An edible insect is in the spotlight as an alternative future food. As the world population has increased, global consumption of conventional meat has increased by approximately 60% within 20 years [1]. For most countries with populations above 10 million in 2011, consumption of meat and fish protein rose over the period of 1961–2011 [2]. Since the world population will continuously increase, there will be a greater difference between demand and supply for every conventional animal product by 2030 [3]. For a sustainable protein supply, in vitro meat or edible insects could be alternative foods [4,5]. Insects have been consumed by humans and traditionally were an integral constituent of human diets in many countries [6]. Despite the hesitation to consume insects in many societies, the global edible insect market size is expected to increase explosively [7].

*Tenebrio molitor* larvae, the mealworm, is a representative edible insect (Figure S1). Nutritionally, mealworms are rich in proteins (17.9% in 100 g of fresh weight), vitamins, and minerals, and they have been widely used as food sources for animals and humans [5,8,9]. One of the characteristics of the mealworms is that they could eat various kinds of feeds regardless of crops, meats, and even fish. This suggests the existence of many pesticide exposure pathways and a high likelihood that pesticide residues are present in mealworms from various feed sources. In controlled studies, pesticides were detected and bioaccumulated in mealworms after they consumed residue-treated crops [10,11]. It is also possible to intentionally spray chemicals on insect farms for pest control [12].

In the United States and South Korea, there are no maximum residue levels (MRLs) for pesticides in edible insects. In the European Union (EU), 423 MRLs were established for terrestrial invertebrate animals, including insects [13]. If the market of edible insects continues to grow, the establishment of a subdivision for insect products and registration of new MRLs is inevitable. Therefore, simultaneous analysis of pesticide multiresidues in edible insects is needed to understand pesticide residue patterns and to rapidly monitor samples obtained from controlled studies or commercial insect farms. In mealworm samples, there have been no reports related to pesticide multiresidue analysis, and only a few studies covering a small number of pesticides have been published [10,11,14,15].

For simultaneous determination of hundreds of pesticides, tandem mass spectrometry and triple quadrupole mass spectrometry (TQ) in particular have been introduced. Conventional single quadrupole mass spectrometry (SQ) requires precise chromatographic separation. Selective ion monitoring (SIM) of SQ cannot distinguish between a target analyte and others with the same mass to charge ratio (*m*/*z*) when their chromatograms are overlapped. TQ provides a multiple reaction monitoring (MRM) mode with a transition pattern between a precursor ion and a product ion. This dramatically reduces the possibility of having the same transition pattern for the target and others. Thus, a highly selective and sensitive analysis is available. TQ is generally coupled with liquid chromatography (LC-MS/MS) or gas chromatography (GC-MS/MS), with which it has been widely used to detect more than a hundred pesticides in food safety areas [16,17,18,19].

Target pesticides may be bound to proteins in mealworm samples due to complex protein folding [20]. Therefore, an effective preparation method for denaturation and elimination of proteins is required. Extraction with organic solvents such as acetonitrile or methanol can denature and precipitate proteins to result in release of pesticides [21,22,23]. The “quick, easy, cheap, effective, rugged, and safe” (QuEChERS) method was introduced for pesticide multiresidue analysis in crops [24]. The QuEChERS procedure comprises an extraction step using a water-acetonitrile mixture for protein denaturation and a solvent partitioning step using various salts for protein precipitation. Therefore, the method as well as modified versions have been successfully applied in protein-rich foods such as legumes [25], livestock [26,27], and fish [19,28]. Organic acids including formic acid or trichloroacetic acid also contribute to protein denaturation [29].

Mealworms contain far more fat (21.9% in 100 g of fresh weight) than conventional livestock such as chicken, egg, beef, and pork (≤9.7%) [8]. Fats may affect chromatographic results, cause severe matrix effects, and produce instrument contamination. Therefore, effective removal of fat during sample preparation is essential. One strategy is a liquid-liquid partitioning between immiscible solvents such as acetonitrile and n-hexane. Many LC-MS/MS-amenable pesticides have higher polarity than non-polar fats. In partitioning, therefore, these pesticides migrate to the polar acetonitrile layer, while fats are entrapped in the non-polar hexane layer. Acetonitrile-hexane partitioning has been applied to soybean samples, which are rich in fat [30]. When fats (especially fatty acids) and proteins remain in the acetonitrile layer, they can be removed by dispersive solid-phase extraction (dSPE) including primary-secondary amines (PSA) or C18. PSA is a weak anion exchanger and effectively removes sugar, fatty acids, organic acids, and lipids [24,31], while C18 is mainly used for eliminating long-chain fatty complex interferences [32,33].

The purpose of this study was to develop a simultaneous multiresidue analysis method for pesticides and related metabolites in mealworms using LC-MS/MS. To effectively detect hundreds of target analytes, a scheduled MRM, where each target is analyzed only in a certain time-window, was adopted. Based on the modified QuEChERS method, we attempted to partition sample extracts with n-hexane to remove fat in mealworms. Using the analytical method, 353 pesticides with LOQ ≤10 μg/kg were validated. The method was applied to real samples obtained from various mealworm farms. This study is the first attempt to simultaneously determine hundreds of pesticide multiresidues in edible insects, which are future food sources.

## 2. Results and Discussion

### 2.1. Optimization of Sample Preparation

After establishment of the MRM transition for each analyte (Table S1) as well as the instrument conditions, sample preparation steps were compared as (1) determination of the number of acetonitrile-hexane partitioning rounds, (2) comparison of sample extract conditions, and (3) comparison of sample cleanup with dSPE sorbents.

#### 2.1.1. Determination of the Number of Acetonitrile-Hexane Partitioning Rounds

Mealworms are rich in fat (21.9% in 100 g of fresh weight) [8] and require effective fat removal to prevent LC-MS/MS from contamination or ion path blocking. During a QuEChERS extraction, most fat is dissolved by acetonitrile and remains in the organic layer. Hexane can easily transport these fats from the acetonitrile layer based on their non-polarity. Because the partitioned hexane waste can contain non-polar target pesticides, additional partitioning with pure acetonitrile can increase the recovery rates of these analytes.

In this study, number of acetonitrile-hexane partitioning rounds (*N* = 1, 2, and 3) was tested. Based on this, recovery and relative standard deviation (RSD) of 353 target analytes are summarized in Table 1. With only one round of partitioning (*N* = 1), more than 74% of the total number of compounds showed excellent recovery (70–120%) and RSD ≤ 20%. An LC-MS/MS-amenable pesticide should have polar chemical moieties to be ionized in the electrospray ionization (ESI) source. Therefore, due to their properties, most target analytes remain in the acetonitrile layer rather than moving to the hexane layer.

**Figure 1:**
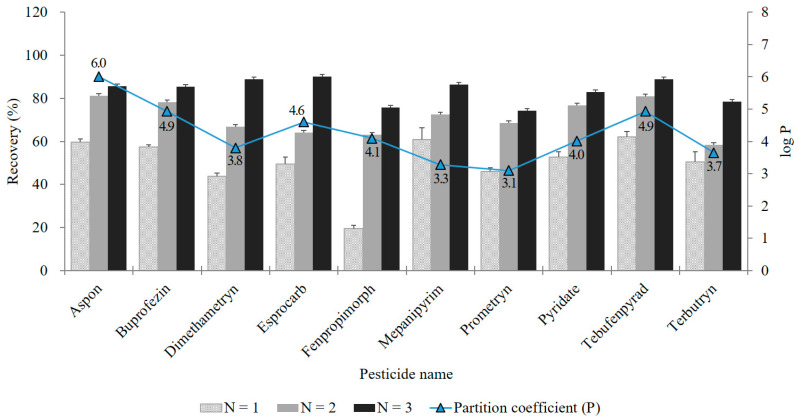
Recovery and partition coefficient (P) [34,35] of 10 representative pesticides that showed a large recovery difference greater than 25% between *N* = 1 and *N* = 3. The error bar is the standard deviation of recovery rate (*n* = 3).

When the number of partitions increased from one to three, the number of pesticides achieving excellent recovery (70–120% with RSD ≤ 20%) increased from 264 (74.8%) to 294 (83.3%) (Table 1). As non-polar pesticides can be distributed limitedly in the hexane layer during acetonitrile-hexane partitioning [30], they were recovered by increasing the number of partitions. Of the target analytes, 26 showed changes in recovery rate between the trials (Table S2), and 10 showed a large recovery difference greater than 25% between *N* = 1 and *N* = 3 (Figure 1), with values of 20–62% for *N* = 1 and 74–90% for *N* = 3. These pesticides have a large partition coefficient (P) with a log P value of 3.1–6.0 and are relatively more non-polar than others [34,35]. Therefore, partitioning with *N* > 1 is essential. To maximize partition efficiencies, *N* = 3 was selected for the best procedure. The reason for the low recovery (<30%) of pesticides at *N* = 3 (Table 1) is dSPE cleanup with PSA rather than acetonitrile-hexane partitioning (Table 2).

#### 2.1.2. Comparison of Sample Extract Conditions

To effectively remove proteins and maximize recovery rates, four QuEChERS extraction combinations were compared. The original QuEChERS and EN 15662 method were established for pesticide multiresidue analysis in crops [24,36]. Recently, modified QuEChERS extraction using acidified acetonitrile containing 0.1% formic acid improved the recovery of some pesticides [16,37]. We compared extraction efficiencies between original and EN 15662 when using acetonitrile or 0.1% formic acid in acetonitrile (Table S3). EN 15662 showed the largest number of pesticides (300; 85.0% of total) with excellent recovery (70–120% with RSD ≤ 20%) when using pure acetonitrile, while the smallest number of pesticides (289; 81.9%) was obtained when using acidified acetonitrile. It seems that citrate buffer in the EN 15662 method helps provide the optimal extraction environment in mealworm samples, while formic acid produces less suitable conditions by lowering the pH. For original salts, there was no significant difference between acetonitrile (294; 83.3%) and acidified acetonitrile (295; 83.6%) extraction (Table S3). As a result, the two combinations showing the best recovery results (“EN 15662 salts + acetonitrile” and “original salts + acidified acetonitrile”) were selected for use in dSPE cleanup conditions.

#### 2.1.3. Comparison of Sample Cleanup with dSPE Sorbents

**Figure 2:**
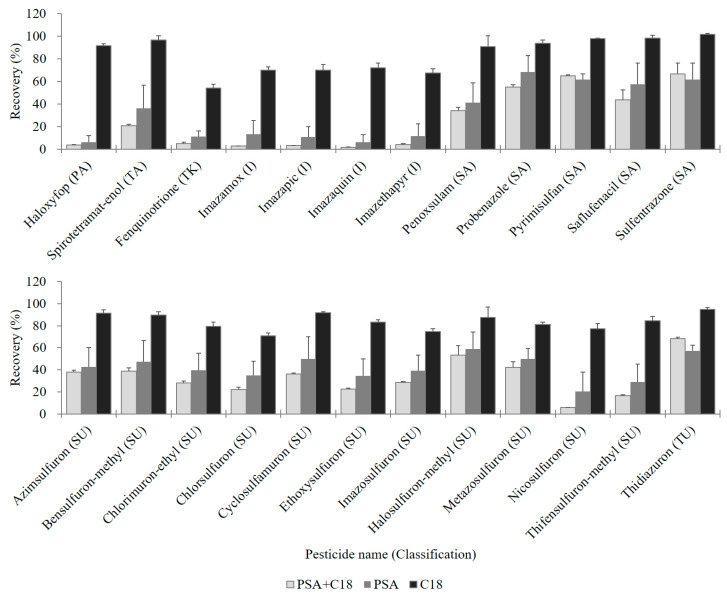
Recoveries of 24 representative pesticides showing a large recovery difference greater than 25% depending on dSPE sorbent combination (PSA + C18, PSA, and C18). PA, propionic acid; TA, tetramic acid; TK, triketone; I, imidazolinone; SA, sulfonamide; SU, sulfonylurea; and TU, thiadiazolylurea. The error bars are the standard deviations of the recovery rates (*n* = 3).

Three types of sorbents PSA + C18 mixture, PSA, and C18 were selected for testing (Table 2 and Figure 2). Under the same sorbent conditions, the combination of “EN 15662 salts + acetonitrile” was always superior to that of “original salts + acidified acetonitrile”, according to the criteria of recovery 70–120% with RSD ≤ 20% (Table 2). Together with the results in Section 2.1.2., we verified that citrate buffer without acid was more effective in the mealworm sample matrices. As a result, the combination of “EN 15662 salts + acetonitrile” was selected for optimized extraction with the proposed method.

When the three dSPE conditions (PSA + C18 mixture, PSA, and C18) were compared under “EN 15662 salts + acetonitrile” extraction based on the criteria (recovery 70–120% with RSD ≤ 20%), C18 sorbent showed better results (325, 92.1% of total) than PSA + C18 (309, 87.5%) and PSA (300, 85.0%) (Table 2).

Among the target analytes, 28 showed different recovery rates between the trials (Table S4), and 24 of them showed a large difference greater than 25% between C18 treatment and the others (Figure 2). These analytes contain propionic acid, tetramic acid, triketone, imidazolinone, sulfonamide, sulfonylurea, or thiadiazolylurea moieties, all of which are acids [35,38]. PSA is a weak anion-exchanger that is advantageous for removing sugars and fatty acids [24,31] but is unsuitable for absorption of target compounds with negative charges. PSA produced high-pH conditions in solution, resulting in these 24 analytes becoming anionic and being caught in the sorbent. This explanation is supported by comparing the recovery results of spirotetramat-enol and its parent compound, spirotetramat. Spirotetramat-enol, a tetramic acid, showed lower recovery (21–36%) under PSA treatment, while non-acidic spirotetramat showed excellent recovery (105–113%) under the proposed conditions (Table S4). This phenomenon has been reported for sulfonylurea [39,40], imidazolinone [41], and other acidic pesticides [42,43].

One the other hand, C18 did not reduce recovery of these 24 analytes (Figure 2), and recovery of all target compounds was greater than 30%, except for TCMTB (Table 2). The reason for the lower recovery of TCMTB (18%) is not the C18, based on a similar low result to that of treatment with PSA only (12%), as shown in Table S4. As C18 sorbent effectively removes non-polar compounds including fats [32,33], it can be used to trap the fats that remain in solution after acetonitrile-hexane partitioning.

From the optimization of sample preparation, the established method comprised three preparation steps: (1) sample extraction with acetonitrile and EN 15662 salts, (2) three (*N* = 3) acetonitrile-hexane partitions, and (3) cleanup with dSPE including C18 sorbent.

### 2.2. Validation of the Analytical Method

Using the established method, validation was conducted according to SANTE/12682/2019 [44]. The evaluation parameters were limit of quantitation (LOQ), linearity of calibration, recovery, and matrix effect.

#### 2.2.1. LOQ

Among the concentrations of various matrix-matched standards, the lowest satisfying signal to noise ratio (*S*/*N*) of 10 or more was selected. The LOQs of all 353 compounds satisfied the criteria at ≤10 μg/kg (Table 3 and Table 4). The sensitivity was sufficient to identify multiple residues in mealworms, according to references of the EU and South Korea legislation that 10 μg/kg is a defualt MRL for pesticides that are not specifically mentioned [13,45].

In the LOQ distribution (Table 4), more than half of the target pesticides (187; 53.0% of total) showed LOQ 1 μg/kg, the most sensitive level in this study. Ninety compounds (25.5%) had LOQ 2.5 μg/kg, and 21.5% of the remaining pesticides had LOQ 5 or 10 μg/kg. Each LOQ was also proven to be reproducible because each RSD of recovery at LOQ was below 20% (Table 4). In conclusion, the sensitivity of all 353 target analytes in this method was sufficient and reasonable for simultaneous determination in mealworm samples.

#### 2.2.2. Linearity of Calibration

Before we determined the linearity of calibration expressed as the correlation coefficient (*r^2^*), the linear range for each analyte was verified (Table 3); the results are summarized in Table 4. Among the 353 compounds, 350 (99.2%) showed a linear range from LOQ to 200 μg/kg. For example, zoxamide with LOQ 1 μg/kg had a linear range of 1–200 μg/kg, and thifluzamide (LOQ 10 μg/kg) had a linear range of 10–200 μg/kg (Table 3). On the other hand, carbendazim, dimethylaminosulfotoluidide (DMST), and methomyl, with LOQ 1 μg/kg, did not show linearity at higher concentrations (≥50 μg/kg) due to signal saturation. These three compounds showed shorter linear ranges from the LOQ to 25 μg/kg; 1–25 μg/kg (Table 3). Within the established linear ranges, all target compounds had excellent linearity with *r^2^* > 0.990 (Table 4). In conclusion, the established analytical method demonstrated a reasonable quantitative relation between concentration and signal.

#### 2.2.3. Recovery

The accuracy and precision of target compounds in the established method were evaluated using average and RSD of recovery (*n* = 6). Two spiked concentrations (a low and a high) were selected according to the linear ranges of the target compounds. If the linear range was from LOQ to 200 μg/kg, the low spiking level was the LOQ (1, 2.5, 5, or 10 μg/kg), and the high level was fixed to 50 μg/kg (Table 3). If the range was from LOQ to 25 μg/kg, the low and high levels were the LOQ (1 μg/kg) and 10 μg/kg, respectively.

Within the spiked range, all 353 analytes showed excellent precision within 2.3% to 19.9% at the low level and within 0.8% to 17.8% at the high level (Table 4). Therefore, this modified QuEChERS method was shown to be rugged and reliable for the target compounds by effectively eliminating protein and fat interferences.

For the recovery rates, 322 (91.2%) analytes at the low and 333 (94.3%) at the high satisfied excellent recovery criteria within 70–120% along with RSD ≤ 20% based on the SANTE/12682/2019 guideline (Table 4) [44]. More than 90% of the analytes showed reasonable accuracies in this study. Some pesticides (32; 9.1% of the total) were not included in the criteria at the low or high level (Table 3) and showed recovery rates of 30–70%. According to the SANTE guideline [44], the method is acceptable (within 30–140% of recovery) for multiresidue analysis, with consistent pesticide recovery rates (RSD ≤ 20%). The pesticide TCMTB showed much lower recovery (~16%) but a consistent RSD (≤18%). Therefore, this method is applicable but limited for screening of TCMTB. It has been reported that TCMTB exhibited higher recovery (70–120%) when using QuEChERS methods in crops and biological samples [46,47]. Thus, further studies of edible insects and livestock are required.

#### 2.2.4. Matrix Effect

The matrix effect is a change in the quantitative relation between concentration and signal, caused by sample matrices. This means that the slope of calibration of a pesticide can be different between a pure solvent and matrix-matched solution. This phenomenon has been demonstrated in LC-MS/MS and GC-MS/MS [48,49], indicating the importance of understanding the matrix effect when solving quantitative problems. In this study, slope of calibration in the matrix-matched solution of the 353 target analytes was compared with that in pure solvent (Table 3). To evaluate the matrix effects, the results were classified into three groups of soft effect (matrix effect within −20% to 0% or 0% to 20%), medium effect (−50% to −20% or 20% and 50%), and strong effect (below −50% or above 50%) [50,51].

More than 94% of the pesticides showed a soft matrix effect (Table 4), with negligible effects in the tested range [51]. In LC-MS/MS, signal suppression by the matrix effect is common [48]. In the present study, however, most of the pesticides were not affected by the matrix, likely due to effective elimination of mealworm matrices. Many proteins and fats causing a severe matrix effect were removed during extraction with organic solvent and salts, acetonitrile-hexane partitioning, and C18 dSPE cleanup. The dilution process during sample preparation also could be helpful. Compared to conventional QuEChERS methods [24,36], 5 to 10 times larger volumes of solvent were used between the extraction and partitioning steps. Dilution decreased the concentration of sample matrices to a level that did not affect the signal. A small proportion of pesticides (5.7%), however, showed a medium or strong matrix effect with this method (Table 4). Thus, a matrix-matched calibration method should be used for correct quantitation.

### 2.3. Application

**Figure 3:**
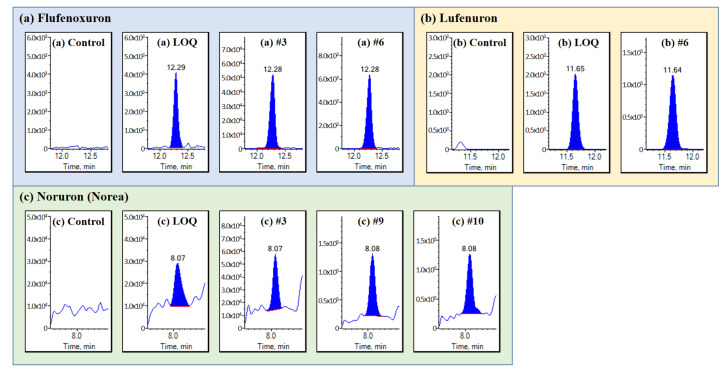
Chromatograms of (**a**) flufenoxuron from the control, LOQ, and mealworm samples (#3 and #6), (**b**) lufenuron from the control, LOQ, mealworm samples (#6), and (**c**) noruron (norea) from the control, LOQ, and agricultural workers (#3, #9, and #10). The multiple reaction monitoring (MRM) transitions in the chromatograms were 489.1 > 158.2 (flufenoxuron), 510.9 > 158.2 (lufenuron), and 223.2 > 67.1 (noruron).

The established method was applied to 11 real samples from commercial mealworm farms (#1 to #11) in South Korea. As shown in Table 5 and Figure 3, the three urea pesticides, flufenoxuron, lufenuron, and noruron (norea), were detected within the range of 1.7−220.7 μg/kg in four samples (#3, #6, #9, and #10). In EU legislation [13], the MRL of lufenuron is 20 μg/kg in terrestrial invertebrate animals including insects, and the MRLs of flufenoxuron and noruron are not established. Houbraken et al. reported that an increased uptake rate by mealworms was observed for pesticides with higher log P [11]. Flufenoxuron and lufenuron have higher log P (4.0 and 5.1) than other LC-MS/MS-amenable pesticides, so they are considered to be accumulated easily in the mealworm’s body [35]. There was no detection of interested pesticides in 30 mealworm samples in South Korea when limited to only five target analytes [15]. The previous study can be powerful in controlled studies with target analytes, while our present study was suitable to obtain wider information of pesticide residue patterns from unknown samples and to help establish the MRL to edible insects.

## 3. Materials and Methods

### 3.1. Reagents

Pesticide standards with high purity (>97%) and stock solutions (1000 μg/mL) were obtained from Dr. Ehrenstorfer (Augsburg, Germany), Wako Pure Chemical Industries (Osaka, Japan), Sigma–Aldrich (St. Louis, MO, USA), ChemService (West Chester, PA, USA), and AccuStandard (New Haven, CT, USA). Ammonium formate (LC−MS grade) was purchased from Sigma−Aldrich. Acetonitrile (HPLC grade), n-hexane (analytical grade), and formic acid (purity; 98–100%) were obtained from Thermo Fisher Scientific (Waltham, MA, USA). Purified water (type I) was prepared in house using an Autwomatic purification system (Wasserlab, Navarra, Spain). The QuEChERS original packet was prepared in house by mixing 1 g NaCl (Merck & Co., Inc., Kenilworth, NJ, USA) and 4 g MgSO_4_ (Sigma-Aldrich) in a 15-mL conical tube. The QuEChERS EN 15662 packet (1 g NaCl, 4 g MgSO_4_, 1 g sodium citrate (Na_3_Citrate · 2H_2_O) and 0.5 g disodium citrate sesquihydrate (Na_2_HCitrate · 1.5H_2_O)), ceramic homogenizers, EMR−lipid^TM^, dSPE kit type I (25 mg PSA, 25 mg C18, and 150 mg MgSO_4_), type II (25 mg PSA and 150 mg MgSO_4_), and type III (25 mg C18 and 150 mg MgSO_4_) were purchased form Agilent Technologies (Santa Clara, CA, USA).

### 3.2. Mealworm Samples

Pesticide-free mealworms for analytical method evaluations were provided by the Industrial Insect Division of the National Institute of Agricultural Sciences in South Korea. Real samples (*n* = 11) were sourced from various mealworm farms in South Korea. All mealworm samples were lyophilized, homogenized with dry ice in a blender, and stored at −20 °C until use.

### 3.3. Working Solutions and Matrix-Matched Standard Solutions

Stock solutions were mixed and diluted with acetonitrile so that the concentration of each analyte was 2.5 μg/mL. The mixed standard solution was serial diluted using acetonitrile to prepare working solutions at concentrations of 1000, 500, 250, 80, 40, 20, 10, 4, 2, 1, 0.4, and 0.2 ng/mL. These solutions were stored at −20 °C until use. Matrix-matched standards were prepared using blank (pesticide-free) mealworms. The blank sample was treated with the same preparation procedures as for the test samples, and the final extract (450 μL) was mixed with the working solution (150 μL). The concentrations of the matrix-matched standards were 20, 10, 5, 2.5, 1, 0.5, 0.25, 0.1, and 0.05 ng/mL, which are equivalent to 200, 100, 50, 25, 10, 5, 2.5, 1, and 0.5 μg analyte per kg mealworm. For the quantitative determination of pesticides, the external standard method without internal standard was selected. Matrix-matched solutions were used for analysis immediately after preparation.

### 3.4. LC-MS/MS Instrumental Conditions

LC-MS/MS analysis was carried out on an AB SCIEX Triple Quad^TM^ 5500 coupled with an Exion LC^TM^ (SCIEX, Redwood City, CA, USA). In UPLC conditions, two mobile phases (A and B) were used, A: 5 mM ammonium formate and 0.1% formic acid in water and B: 5 mM ammonium formate and 0.1% formic acid in methanol. Column oven and sample tray temperatures were 40 °C and 15 °C, respectively. The gradient condition was started at 5% of mobile phase B for 0.2 min, ramped by 50% for 0.3 min, increased by 90% for 9 min, increased by 98% for 4 min, and maintained at 98% for 3.5 min. To analyze the next sample, B% was sharply decreased by 5% for 0.1 min and maintained for 2.9 min to achieve equilibrium. The total separation time was 20 min. Chromatographic separation was performed using a Halo C18 (2.1 × 150 mm, 2.7 μm) column (Advanced Materials Technology, Wilmington, DE, USA), and the injection volume was 2 μL. In the tandem MS condition, the ionization source was the Turbo V^TM^ (SCIEX), and an electrospray ionization (ESI) probe capable of positive-negative switching during sample analysis was utilized. The pressures of the curtain gas (CUR), collision gas (CAD), and ion source gases 1 and 2 (GS1 and GS2) were 25, 10, 50, and 50 psi, respectively. The source temperature was 550 °C, and the ion spray voltage (IS) was +5500 V for positive mode and −4500 V for negative mode. The scheduled MRM was applied to all target pesticides, and the quantitative results of MRM data were processed by the MultiQuant^TM^ 3.0.2 (version number: 3.0.8664.0, SCIEX).

### 3.5. Comparison of Preparation Procedures

Before evaluation of preparation procedures, recovery samples were prepared. Blank mealworm samples were verified to be free from target pesticides using previous QuEChERS methods [24,36]. Homogenized blank mealworm samples (5 g) were put into a 50 mL conical tube and treated with 100 μL of 2500 ng/mL working solutions so that the concentration of each target pesticide in the sample was 25 μg/kg.

To verify the partition efficiency using polar acetonitrile and non-polar n-hexane, pesticide-spiked samples (25 μg/kg) were soaked in 7 mL water for 15 min. Each sample was extracted with 12.5 mL acetonitrile and centrifuged after the QuEChERS original packet (1 g NaCl and 4 g MgSO_4_) were added to the tube. The acetonitrile layer (6 mL) was transferred into a 15-mL tube, mixed with 4 mL n-hexane, and centrifuged. The lower layer (acetonitrile) was transferred into a new tube, and the remaining upper layer (hexane) was discarded (*N* = 1) or partitioned with n-hexane-saturated acetonitrile (6 mL) once (*N* = 2) or twice (*N* = 3). For each trial, partitioned acetonitrile layers were pooled and treated with dSPE (25 mg PSA and 150 mg MgSO_4_). After cleanup, 450 μL of sample was matrix-matched with 150 μL acetonitrile to evaluate recoveries of target pesticides.

To evaluate extraction efficiencies between extraction solvents and QuEChERS salts, the soaked mealworm samples (25 μg/kg) were extracted with 12.5 mL acetonitrile or 0.1% formic acid acetonitrile. Each sample was treated with the QuEChERS original packet (1 g NaCl and 4 g MgSO_4_) or EN 15662 packet (1 g NaCl, 4 g MgSO_4_, 1 g Na_3_Citrate · 2H_2_O, and 0.5 g Na_2_HCitrate · 1.5H_2_O). The organic layer (6 mL) was partitioned with 4 mL n-hexane. The lower layer (acetonitrile) was transferred to a new tube, and the remaining upper layer (hexane) was further partitioned twice with n-hexane-saturated acetonitrile (6 mL). The combined acetonitrile layers were cleaned with dSPE (25 mg PSA and 150 mg MgSO_4_). The sample (450 μL) was matrix-matched with 150 μL acetonitrile to evaluate recoveries of target pesticides.

To compare cleanup efficiencies with various cleanup sorbents, the soaked mealworm samples (25 μg/kg) were extracted with 12.5 mL acetonitrile and EN 15662 packet, and the upper layer was partitioned with n-hexane (4 mL). The remaining upper layer (hexane) was partitioned twice with n-hexane-saturated acetonitrile (6 mL). Each sample was treated with dSPE type I (25 mg PSA, 25 mg C18, and 150 mg MgSO_4_), type II (25 mg PSA and 150 mg MgSO_4_), and type III (25 mg C18 and 150 mg MgSO_4_). After cleanup, 450 μL of sample was matrix-matched with 150 μL acetonitrile to evaluate recoveries of target pesticides.

### 3.6. Established Sample Preparation Procedures

Five grams of homogenized mealworms were transferred into a 50-mL conical tube, and 7 mL water was added for 15 min. After addition of 12.5 mL acetonitrile and two ceramic homogenizers, the sample was shaken at 1300 rpm for 2 min using a Geno/Grinder (SPEX SamplePrep, Metuchen, NJ, USA), and the QuEChERS EN 15662 packet (1 g NaCl, 4 g MgSO_4_, 1 g Na_3_Citrate · 2H_2_O, and 0.5 g Na_2_HCitrate · 1.5H_2_O) was poured into the tube. After shaking at 1300 rpm for 1 min, the sample was centrifuged at 3500 rpm for 5 min using Combi-514R (Hanil Science Co., LTD., Incheon, South Korea). The organic layer (6 mL) was transferred into a 15-mL tube, mixed with 4 mL n-hexane, and centrifuged for 3500 rpm for 5 min. The lower layer (acetonitrile) was transferred into a new tube, and the remaining upper layer (hexane) was partitioned twice with n-hexane-saturated acetonitrile (6 mL). The acetonitrile layers were combined, and 1 mL of the extract was placed in the dSPE kit containing 25 mg C18 and 150 mg MgSO_4_. The kit was mixed for 1 min and centrifuged at 12,000 rpm for 5 min using Combi-514R. The upper layer (450 μL) was matrix-matching with 150 μL acetonitrile, and 2 µL of the final extract was injected into the LC-MS/MS.

### 3.7. Method Validation and Matrix Effect

For each target analyte, the LOQ was determined as the minimum concentration providing an *S*/*N* of 10 on the chromatogram as well as a reasonable recovery precision (RSD ≤ 20%). The calibration curve was obtained from matrix-matched standards, and the linearity of calibration was expressed as correlation coefficient (*r^2^*) at the weighting factor *1/x*. The recovery was evaluated at two fortification levels (the lower one at LOQ: 1, 2.5, 5, or 10 mg/kg, and the higher one: 10 or 50 mg/kg). In the recovery test, 100 μL working solution was spiked into 5 g samples, and the samples were prepared with the established procedures. Each chromatographic area from the sample was interpolated into the matrix-matched standard calibration curve to calculate the recovery rate. For each fortification level, the accuracy was expressed as the average of recovery rates (*n* = 6), and the precision was expressed by its RSD. The matrix effect for each analyte was evaluated by comparing the slope of calibration from the matrix-matched standard solution with that from the standard in pure solvent. The degree of the matrix effect can be expressed using Equation (1).

(1) $$\mathrm{Matrix}\;\mathrm{effect},\;\%=\left(\frac{\mathrm{Slope}\;\mathrm{of}\;\mathrm{calibration}\;\mathrm{in}\;\mathrm{matrix}\;\mathrm{matched}\;\mathrm{solution}}{\mathrm{Slope}\;\mathrm{of}\;\mathrm{calibration}\;\mathrm{in}\;\mathrm{pure}\;\mathrm{solvent}}-1\right)\times 100$$

## 4. Conclusions

Using LC-MS/MS, a novel multiresidual method for simultaneous analysis of pesticides and related metabolites in mealworm samples was developed and validated. The scheduled MRMs for 353 analytes were established for a high-throughput triple quadrupole mass spectrometer. During mealworm sample preparation, fat elimination was successful without loss of target analytes through several rounds of acetonitrile-hexane partitioning. C18 sorbent dSPE showed the highest cleanup efficiencies for all target analytes, while dSPE including PSA caught some compounds having anionic moieties. The established analytical method was validated based on four parameters: LOQ, linearity of calibration, recovery, and matrix effect. Target analytes satisfied the sensitivities and quantitative properties required by the EU and South Korea legislation, and the SANTE guideline. For the first time, simultaneous determination of hundreds of multiresidues on 11 real mealworm samples was conducted, and the established method was proven to be applicable by positive detection of three urea pesticides (flufenoxuron, lufenuron, and noruron) in four samples.

## Figures and Tables

**Table 1:** Number of acetonitrile-hexane partitioning rounds (*N* = 1, 2, and 3) and distribution of recovery results for 353 target pesticides. Each partitioning round was conducted with acetonitrile extraction using “quick, easy, cheap, effective, rugged, and safe” method (QuEChERS) original salts (NaCl and MgSO_4_) and dispersive solid-phase extraction (dSPE) cleanup using primary-secondary amines (PSA) sorbent. RSD—relative standard deviation.

Recovery (%) at 25 μg/kg	RSD (%) *n* = 3	No. of Pesticides (%)		
		*N* = 1	*N* = 2	*N* = 3
<10	>0	6 (1.7)	6 (1.7)	7 (2.0)
10 to 30	≤20	12 (3.4)	13 (3.7)	9 (2.5)
	>20	1 (0.3)	0 (0.0)	1 (0.3)
30 to 70	≤20	55 (15.6)	31 (8.8)	30 (8.5)
	>20	3 (0.8)	3 (0.8)	1 (0.3)
70 to 120	≤20	264 (74.8)	290 (82.2)	294 (83.3)
	>20	9 (2.5)	7 (2.0)	8 (2.3)
>120	≤20	2 (0.6)	2 (0.6)	3 (0.8)
	>20	0 (0.0)	0 (0.0)	0 (0.0)
nd ^1^		1 (0.3)	1 (0.3)	0 (0.0)
Sum		353 (100)	353 (100)	353 (100)

^1^ Not detected.

**Table 2:** Comparison of three dSPE sorbent combinations (PSA + C18, PSA, and C18) at two sample preparation conditions and recovery results for 353 target pesticides. In each preparation, acetonitrile-hexane partitioning (*N* = 3) was carried out.

Recovery (%) at 25 μg/kg	RSD (%) *n* = 3	No. of Pesticides (%) under Detailed Sample Preparation Conditions					
		0.1% Formic Acid in Acetonitrile (12.5 mL) QuEChERS Original Salts ^1^			Acetonitrile (12.5 mL) QuEChERS EN 15662 Salts ^2^		
		PSA + C18	PSA	C18	PSA + C18	PSA	C18
<10	>0	7 (2.0)	6 (1.7)	0 (0.0)	7 (2.0)	2 (0.6)	0 (0.0)
10 to 30	≤20	12 (3.4)	5 (1.4)	1 (0.3)	7 (2.0)	1 (0.3)	0 (0.0)
	>20	0 (0.0)	5 (1.4)	1 (0.3)	1 (0.3)	6 (1.7)	1 (0.3)
30 to 70	≤20	21 (5.9)	20 (5.7)	26 (7.4)	23 (6.5)	17 (4.8)	21 (5.9)
	>20	1 (0.3)	3 (0.8)	0 (0.0)	1 (0.3)	15 (4.2)	0 (0.0)
70 to 120	≤20	305 (86.4)	295 (83.6)	318 (90.1)	309 (87.5)	300 (85.0)	325 (92.1)
	>20	2 (0.6)	12 (3.4)	0 (0.0)	1 (0.3)	10 (2.8)	0 (0.0)
>120	≤20	5 (1.4)	6 (1.7)	7 (2.0)	4 (1.1)	2 (0.6)	6 (1.7)
	>20	0 (0.0)	0 (0.0)	0 (0.0)	0 (0.0)	0 (0.0)	0 (0.0)
nd ^3^		0 (0.0)	1 (0.3)	0 (0.0)	0 (0.0)	0 (0.0)	0 (0.0)
Sum		353 (100)	353 (100)	353 (100)	353 (100)	353 (100)	353 (100)

^1^ NaCl (1 g), MgSO_4_ (4 g). ^2^ NaCl (1 g), MgSO_4_ (4 g), Na_3_Citrate · 2H_2_O (1 g), Na_2_HCitrate · 1.5H_2_O (0.5 g). ^3^ Not detected.

**Table 3:** Limit of quantitation (LOQ), linearity of calibration (*r^2^*), recovery, and matrix effect validation results for the 353 target pesticides.

No.	Compound Name	LOQ (μg/kg)	*r^2^*	Linear Range (μg/kg)	Recovery						ME ^2^ (%)
					Low			High			
					Conc. ^1^ (μg/kg)	Value (%)	RSD (%)	Conc. (μg/kg)	Value (%)	RSD (%)	
1	Abamectin	10	0.9923	10–200	10	108.1	13.9	50	112.5	8.3	11.8
2	Acetamiprid	1	0.9975	1–200	1	90.2	12.2	50	103.8	3.2	5.8
3	Acibenzolar-S-methyl	5	0.9991	5–200	5	100.5	15.8	50	83.3	4.8	4.9
4	Alachlor	5	0.9993	5–200	5	90.2	14.1	50	93.5	2.7	−6.8
5	Aldicarb sulfone	1	0.9993	1–200	1	87.4	18.7	50	101.5	2.0	−0.7
6	Aldicarb sulfoxide	1	0.9999	1–200	1	82.8	12.2	50	94.7	1.4	−11.3
7	Allethrin	10	0.9971	10–200	10	85.5	13.2	50	84.7	3.2	−9.2
8	Ametoctradin	2.5	0.9996	2.5–200	2.5	80.4	8.9	50	80.5	3.2	−4.7
9	Ametryn	10	0.9982	10–200	10	65.8	4.4	50	75.0	7.1	−6.5
10	Anilofos	1	0.9980	1–200	1	102.9	16.5	50	94.2	10.2	−6.5
11	Aramite	1	0.9997	1–200	1	89.1	15.9	50	94.4	4.7	−24.0
12	Aspon	1	0.9998	1–200	1	87.7	4.2	50	89.5	1.9	−10.6
13	Atrazine	1	0.9999	1–200	1	89.5	6.6	50	85.6	1.2	1.9
14	Azaconazole	1	0.9983	1–200	1	76.9	8.3	50	87.6	3.5	5.3
15	Azimsulfuron	2.5	0.9992	2.5–200	2.5	84.9	14.1	50	92.8	4.2	2.9
16	Azinphos-ethyl	2.5	0.9925	2.5–200	2.5	106.2	8.6	50	113.7	9.8	−21.3
17	Azinphos-methyl	2.5	0.9931	2.5–200	2.5	95.3	14.5	50	107.8	8.1	−17.8
18	Azoxystrobin	1	0.9950	1–200	1	93.5	9.8	50	105.6	3.6	−2.6
19	Benalaxyl	1	0.9980	1–200	1	91.5	11.3	50	97.5	5.1	−13.5
20	Bendiocarb	2.5	0.9982	2.5–200	2.5	95.4	5.8	50	95.4	4.5	3.3
21	Benodanil	1	0.9997	1–200	1	92.4	7.3	50	96.8	2.8	1.7
22	Benoxacor	2.5	0.9988	2.5–200	2.5	96.7	14.8	50	96.6	3.8	3.2
23	Bensulfuron-methyl	1	0.9994	1–200	1	88.7	15.6	50	94.6	4.5	7.7
24	Bensulide	2.5	0.9942	2.5–200	2.5	73.0	16.4	50	111.4	6.9	−22.6
25	Benthiavalicarb-isopropyl	1	0.9994	1–200	1	107.1	11.8	50	95.6	2.7	1.8
26	Benzobicyclon	1	0.9993	1–200	1	118.0	9.1	50	94.6	3.4	15.6
27	Benzoximate	5	0.9959	5–200	5	69.1	9.5	50	87.5	9.3	−6.9
28	Benzoylprop-ethyl	2.5	0.9997	2.5–200	2.5	103.7	8.7	50	96.3	7.2	−4.5
29	Bitertanol	5	0.9976	5–200	5	96.4	13.3	50	95.7	7.2	10.4
30	Bixafen	1	0.9974	1–200	1	109.6	18.1	50	97.1	9.4	−8.0
31	Boscalid	5	0.9992	5–200	5	89.0	7.3	50	96.1	3.1	5.6
32	Broflanilide	10	0.9965	10–200	10	111.6	16.9	50	103.7	5.7	11.5
33	Broflanilide_DM-8007	1	0.9994	1–200	1	114.8	14.9	50	97.1	6.9	−0.2
34	Broflanilide_S(PFP-OH)-8007	10	0.9977	10–200	10	97.6	14.5	50	106.1	7.6	−4.7
35	Bromacil	2.5	0.9978	2.5–200	2.5	91.6	9.4	50	96.7	5.2	4.4
36	Bromobutide	2.5	0.9983	2.5–200	2.5	99.1	15.8	50	99.0	9.2	−7.1
37	Bromuconazole	5	0.9977	5–200	5	78.1	12.5	50	85.2	7.9	6.1
38	Bupirimate	1	0.9969	1–200	1	86.5	18.1	50	91.4	10.7	−3.4
39	Buprofezin	2.5	0.9998	2.5–200	2.5	73.2	8.5	50	72.1	6.4	−26.5
40	Butocarboxim	10	0.9958	10–200	10	116.8	8.4	50	106.4	6.9	−4.5
41	Cadusafos	1	0.9989	1–200	1	73.5	6.8	50	88.1	2.0	2.8
42	Cafenstrole	2.5	0.9997	2.5–200	2.5	99.2	13.1	50	106.7	8.6	0.4
43	Carbaryl	1	0.9988	1–200	1	83.3	19.2	50	89.8	4.3	4.1
44	Carbendazim	1	0.9999	1–25	1	46.6	14.7	10	47.5	6.6	−2.3
45	Carbetamide	2.5	0.9958	2.5–200	2.5	75.2	10.0	50	95.4	5.0	12.6
46	Carbofuran	10	0.9902	10–200	10	79.4	7.3	50	115.6	4.7	−25.4
47	Carbofuran-3-hydroxy	5	0.9989	5–200	5	84.7	17.8	50	95.6	8.3	10.5
48	Carboxin	1	0.9986	1–200	1	88.9	11.4	50	88.3	3.4	−8.6
49	Carfentrazone-ethyl	10	0.9994	10–200	10	91.8	19.9	50	93.6	4.7	1.0
50	Carpropamid	5	0.9976	5–200	5	86.9	14.8	50	98.4	3.7	−1.4
51	Chlorantraniliprole	1	0.9994	1–200	1	100.6	17.1	50	96.8	4.2	9.1
52	Chlorbenzuron	10	0.9950	10–200	10	84.9	19.7	50	95.0	9.1	5.6
53	Chlorfenvinphos	2.5	0.9989	2.5–200	2.5	90.3	8.0	50	96.0	6.2	−18.2
54	Chlorimuron-ethyl	1	0.9995	1–200	1	89.8	11.1	50	92.3	3.5	8.4
55	Chlorotoluron	1	0.9998	1–200	1	94.3	8.5	50	92.7	3.2	−7.2
56	Chloroxuron	1	0.9966	1–200	1	103.3	16.9	50	97.0	6.8	−2.1
57	Chlorpyrifos	2.5	0.9997	2.5–200	2.5	86.6	12.8	50	83.9	3.9	−14.9
58	Chlorsulfuron	5	0.9965	5–200	5	61.5	10.9	50	88.9	15.9	−2.4
59	Chromafenozide	1	0.9970	1–200	1	96.5	9.7	50	97.3	8.1	−2.0
60	Clethodim	1	0.9975	1–200	1	93.8	19.2	50	82.3	7.1	2.1
61	Clofentezine	1	0.9985	1–200	1	64.9	18.1	50	55.0	7.9	4.7
62	Clomazone	2.5	0.9980	2.5–200	2.5	94.1	8.0	50	91.4	5.0	14.7
63	Clothianidin	5	0.9979	5–200	5	83.3	14.3	50	88.8	7.1	9.5
64	Coumaphos	1	0.9982	1–200	1	82.1	8.4	50	92.5	2.7	4.6
65	Crotoxyphos	10	0.9991	10–200	10	42.9	11.4	50	68.2	8.5	2.6
66	Crufomate	2.5	0.9989	2.5–200	2.5	92.3	5.2	50	98.0	4.9	−9.7
67	Cyanazine	2.5	0.9993	2.5–200	2.5	100.1	11.1	50	99.1	4.1	10.5
68	Cyazofamid	1	0.9978	1–200	1	99.3	7.3	50	97.7	4.4	8.8
69	Cyclosulfamuron	1	0.9950	1–200	1	92.1	15.6	50	100.5	2.7	−2.8
70	Cyflufenamid	1	0.9966	1–200	1	100.5	18.8	50	103.6	3.2	−14.3
71	Cyhalofop-butyl	10	0.9980	10–200	10	90.9	11.1	50	90.2	13.3	8.5
72	Cymoxanil	2.5	0.9988	2.5–200	2.5	89.0	13.0	50	94.9	5.3	13.0
73	Cyprazine	1	0.9998	1–200	1	95.2	6.3	50	86.6	4.2	−3.3
74	Cyprodinil	10	0.9995	10–200	10	43.5	7.1	50	55.2	5.7	−4.1
75	Daimuron	1	0.9993	1–200	1	83.5	11.3	50	97.2	4.4	7.9
76	Demeton-S	1	0.9961	1–200	1	91.3	13.3	50	99.6	6.6	5.7
77	Demeton-S-methyl	5	0.9976	5–200	5	81.2	9.3	50	98.6	8.3	8.9
78	Demeton-S-methyl sulfone	2.5	0.9987	2.5–200	2.5	71.2	11.0	50	94.5	7.0	7.5
79	Demeton-S-methyl sulfoxide	2.5	0.9995	2.5–200	2.5	79.9	7.9	50	87.5	3.8	−1.2
80	Desmetryn	10	0.9990	10–200	10	70.1	3.4	50	74.0	2.7	0.8
81	Diazinon	2.5	0.9993	2.5–200	2.5	89.3	10.4	50	88.4	3.7	−3.2
82	Diclobutrazol	10	0.9987	10–200	10	86.6	7.0	50	88.2	6.5	−9.1
83	Dicrotophos	5	0.9999	5–200	5	52.6	18.4	50	73.8	8.7	−0.2
84	Diethatyl-ethyl	1	0.9992	1–200	1	107.5	9.8	50	97.8	7.2	0.7
85	Diethofencarb	1	0.9993	1–200	1	111.9	10.9	50	97.6	5.2	16.6
86	Difenoconazole	1	0.9996	1–200	1	108.5	17.4	50	88.5	2.1	−1.7
87	Diflubenzuron	1	0.9993	1–200	1	103.9	18.7	50	98.5	4.5	2.6
88	Diflufenican	1	0.9996	1–200	1	86.3	11.9	50	94.1	3.6	10.1
89	Dimepiperate	5	0.9937	5–200	5	97.1	9.1	50	91.1	11.5	−9.7
90	Dimethachlor	2.5	0.9996	2.5–200	2.5	91.2	14.4	50	97.6	2.6	1.9
91	Dimethametryn	1	0.9990	1–200	1	62.0	8.6	50	69.1	3.6	−2.9
92	Dimethenamide	2.5	0.9987	2.5–200	2.5	83.2	10.8	50	96.1	3.2	−1.7
93	Dimethoate	1	0.9979	1–200	1	98.3	16.7	50	100.4	7.3	4.4
94	Dimethomorph	1	0.9993	1–200	1	95.1	18.7	50	101.2	2.4	7.5
95	Dimethylaminosulfotoluidide (DMST)	1	0.9989	1–25	1	94.2	15.3	10	88.9	4.7	6.0
96	Dimethylvinphos (E)	2.5	0.9989	2.5–200	2.5	100.6	15.7	50	95.9	6.0	2.3
97	Dimethylvinphos (Z)	10	0.9996	10–200	10	63.3	6.2	50	84.0	4.3	−0.2
98	Diniconazole	5	0.9997	5–200	5	57.0	16.7	50	90.6	4.2	−19.3
99	Dinotefuran	5	0.9987	5–200	5	104.8	16.4	50	103.1	5.8	−21.9
100	Diphenamid	1	0.9958	1–200	1	86.0	6.2	50	103.8	3.7	0.7
101	Dithiopyr	1	0.9997	1–200	1	86.5	12.3	50	98.2	3.7	4.8
102	Diuron	1	0.9996	1–200	1	94.2	11.6	50	92.2	3.0	4.5
103	Edifenphos	10	0.9932	10–200	10	71.4	6.3	50	85.7	7.9	−6.9
104	Esprocarb	2.5	0.9992	2.5–200	2.5	83.4	10.3	50	83.1	5.9	−24.4
105	Etaconazole	2.5	0.9981	2.5–200	2.5	92.5	18.1	50	94.6	5.0	−4.1
106	Ethaboxam	2.5	0.9978	2.5–200	2.5	119.8	7.3	50	99.7	2.1	12.0
107	Ethiofencarb	1	0.9992	1–200	1	92.3	15.4	50	93.6	5.1	3.3
108	Ethoprophos	1	0.9933	1–200	1	83.7	16.1	50	100.6	6.3	0.2
109	Ethoxysulfuron	2.5	0.9973	2.5–200	2.5	76.3	14.8	50	87.9	4.4	−10.4
110	Etofenprox	1	0.9996	1–200	1	81.5	13.3	50	75.2	1.4	−10.8
111	Etoxazole	1	0.9968	1–200	1	74.9	2.3	50	88.9	2.0	−12.5
112	Etrimfos	2.5	0.9994	2.5–200	2.5	95.0	11.7	50	94.8	3.7	−4.3
113	Famoxadone	5	0.9959	5–200	5	95.4	18.7	50	102.7	5.6	0.4
114	Fenamiphos	1	0.9997	1–200	1	97.8	13.4	50	93.0	6.1	−1.7
115	Fenamiphos sulfone	2.5	0.9949	2.5–200	2.5	102.9	19.6	50	86.4	7.4	6.5
116	Fenamiphos sulfoxide	1	0.9994	1–200	1	79.9	15.1	50	85.1	5.3	12.3
117	Fenazaquin	1	0.9999	1–200	1	57.2	8.3	50	61.2	2.8	–39.7
118	Fenbuconazole	2.5	0.9979	2.5–200	2.5	95.9	17.0	50	98.4	5.8	2.2
119	Fenfuram	1	0.9982	1–200	1	89.8	14.3	50	105.1	4.6	–2.1
120	Fenhexamid	10	0.9991	10–200	10	71.3	12.7	50	93.1	5.1	5.8
121	Fenobucarb	1	0.9997	1–200	1	94.0	7.2	50	92.8	5.1	–3.2
122	Fenothiocarb	1	0.9996	1–200	1	100.4	15.0	50	92.8	2.7	–0.1
123	Fenoxanil	2.5	0.9994	2.5–200	2.5	108.5	17.4	50	97.1	5.0	–1.1
124	Fenoxaprop-ethyl	1	0.9988	1–200	1	84.8	7.6	50	91.4	3.7	1.0
125	Fenoxycarb	5	0.9996	5–200	5	87.4	7.5	50	100.5	5.9	–1.6
126	Fenpropathrin	1	0.9997	1–200	1	71.4	15.8	50	90.7	2.7	13.6
127	Fenpropimorph	1	0.9989	1–200	1	88.6	16.3	50	83.6	5.6	–0.4
128	Fenpyroximate	1	0.9999	1–200	1	84.0	6.3	50	85.0	1.4	16.4
129	Fenquinotrione	10	0.9998	10–200	10	47.3	18.6	50	49.7	8.1	7.3
130	Fenthion	2.5	0.9972	2.5–200	2.5	75.7	12.3	50	91.6	4.0	2.3
131	Fenthion oxon	1	0.9977	1–200	1	97.2	2.9	50	93.0	4.5	2.7
132	Fenthion oxon sulfoxide	2.5	0.9994	2.5–200	2.5	73.5	15.8	50	81.3	2.4	3.2
133	Fenthion sulfone	1	0.9974	1–200	1	105.9	19.2	50	101.6	3.6	17.8
134	Fenthion sulfoxide	1	0.9988	1–200	1	72.5	19.5	50	100.1	5.0	4.3
135	Fentrazamide	1	0.9980	1–200	1	103.2	11.8	50	99.2	8.3	12.4
136	Ferimzone	5	0.9991	5–200	5	41.0	18.3	50	67.0	1.4	–1.9
137	Fipronil	1	0.9995	1–200	1	99.9	5.1	50	97.8	1.2	24.7
138	Fipronil sulfone	1	0.9998	1–200	1	97.2	3.5	50	100.7	2.8	4.8
139	Flamprop-isopropyl	1	0.9989	1–200	1	89.8	16.0	50	99.9	5.8	–1.3
140	Flonicamid	2.5	0.9982	2.5–200	2.5	89.6	12.0	50	100.3	5.0	–11.2
141	Fluacrypyrim	1	0.9983	1–200	1	85.1	18.9	50	99.9	6.6	–9.6
142	Fluazinam	1	0.9999	1–200	1	80.3	2.5	50	86.1	0.8	12.8
143	Flucetosulfuron	10	0.9957	10–200	10	59.0	8.9	50	75.2	6.9	0.7
144	Fludioxonil	1	0.9997	1–200	1	90.4	8.1	50	95.3	2.9	16.5
145	Flufenacet	1	0.9944	1–200	1	101.3	11.2	50	105.6	10.9	1.8
146	Flufenoxuron	1	0.9999	1–200	1	88.9	5.8	50	93.0	3.9	11.0
147	Fluometuron	5	0.9994	5–200	5	87.2	19.4	50	96.2	3.9	5.5
148	Fluopicolide	1	0.9974	1–200	1	86.7	9.7	50	99.0	3.4	3.6
149	Fluopyram	1	0.9983	1–200	1	99.6	11.5	50	101.7	9.0	–0.3
150	Flupyradifuron	1	0.9944	1–200	1	95.6	16.0	50	104.2	3.2	9.9
151	Fluquinconazole	2.5	0.9983	2.5–200	2.5	101.8	14.9	50	93.0	4.0	–4.5
152	Fluridone	1	0.9982	1–200	1	86.5	9.6	50	98.6	6.9	–3.1
153	Flurochloridone	2.5	0.9940	2.5–200	2.5	88.5	17.6	50	96.8	7.9	1.0
154	Flurtamone	1	0.9932	1–200	1	98.7	11.0	50	101.6	7.7	–4.2
155	Flusilazole	1	0.9994	1–200	1	92.8	17.2	50	92.7	5.5	–8.6
156	Fluthiacet-methyl	10	0.9964	10–200	10	71.6	12.4	50	76.7	15.6	6.6
157	Flutianil	1	0.9983	1–200	1	95.7	15.3	50	95.7	5.2	5.6
158	Flutolanil	1	0.9943	1–200	1	90.8	11.1	50	102.2	4.8	–0.8
159	Flutriafol	1	0.9990	1–200	1	101.2	13.0	50	98.0	3.8	–0.4
160	Fluxapyroxad	1	0.9977	1–200	1	97.3	12.6	50	104.5	4.7	–5.3
161	Forchlorfenuron	1	0.9979	1–200	1	81.0	17.3	50	87.9	3.0	2.6
162	Fosthiazate	1	0.9979	1–200	1	88.0	7.8	50	99.6	1.9	–1.5
163	Halosulfuron-methyl	2.5	0.9959	2.5–200	2.5	88.1	13.9	50	90.4	8.7	–2.7
164	Haloxyfop	2.5	0.9991	2.5–200	2.5	94.8	11.5	50	84.9	2.9	11.4
165	Heptenophos	10	0.9997	10–200	10	69.0	5.2	50	83.8	5.4	–7.2
166	Hexaconazole	10	0.9954	10–200	10	80.6	12.6	50	87.6	3.3	–14.8
167	Hexazinone	1	0.9948	1–200	1	77.8	8.6	50	99.7	6.9	1.1
168	Hexythiazox	1	0.9990	1–200	1	81.5	17.9	50	80.6	3.1	–0.4
169	Imazalil	5	0.9997	5–200	5	78.1	7.0	50	87.7	2.6	–15.8
170	Imazamox	10	0.9988	10–200	10	62.9	7.9	50	68.2	6.4	8.3
171	Imazapic	2.5	0.9987	2.5–200	2.5	72.5	7.2	50	69.1	3.6	5.3
172	Imazaquin	2.5	0.9988	2.5–200	2.5	84.8	12.9	50	70.7	4.5	6.5
173	Imazethapyr	1	0.9993	1–200	1	100.6	10.5	50	73.2	3.3	8.9
174	Imazosulfuron	2.5	0.9989	2.5–200	2.5	92.1	10.1	50	82.7	3.8	7.4
175	Imicyafos	10	0.9918	10–200	10	102.7	9.9	50	95.1	7.0	–4.2
176	Imidacloprid	1	0.9994	1–200	1	94.4	19.7	50	94.3	9.6	5.9
177	Inabenfide	1	0.9991	1–200	1	105.8	6.2	50	86.9	2.1	80.9
178	Indanofan	5	0.9996	5–200	5	95.9	13.7	50	92.9	4.1	4.6
179	Ipconazole	1	0.9992	1–200	1	104.6	19.8	50	85.9	3.4	–11.5
180	Iprobenfos	1	0.9993	1–200	1	76.8	17.5	50	95.0	4.4	–0.7
181	Iprovalicarb	1	0.9998	1–200	1	90.9	14.3	50	99.3	5.3	1.1
182	Isoprocarb	1	0.9999	1–200	1	97.7	12.5	50	93.8	4.7	3.7
183	Isoprothiolane	2.5	0.9989	2.5–200	2.5	94.0	14.6	50	89.1	11.7	32.2
184	Isoproturon	1	0.9998	1–200	1	97.0	7.2	50	93.0	2.2	1.8
185	Isopyrazam	2.5	0.9992	2.5–200	2.5	101.3	6.7	50	95.5	2.2	–0.7
186	Isoxaben	1	0.9987	1–200	1	99.5	10.8	50	101.9	4.3	10.5
187	Isoxathion	5	0.9980	5–200	5	74.9	4.2	50	93.3	5.0	–14.0
188	Kresoxim-methyl	10	0.9983	10–200	10	77.2	15.5	50	93.1	15.8	–13.9
189	Lenacil	1	0.9998	1–200	1	86.4	16.8	50	85.3	2.8	3.9
190	Linuron	2.5	0.9985	2.5–200	2.5	86.5	11.6	50	94.6	3.8	–0.7
191	Lufenuron	2.5	0.9993	2.5–200	2.5	105.9	15.0	50	95.8	5.2	–35.5
192	Malaoxon	10	0.9991	10–200	10	40.6	14.7	50	65.4	12.9	1.6
193	Malathion	1	0.9915	1–200	1	94.8	16.0	50	94.0	4.6	–0.1
194	Mandipropamid	1	0.9992	1–200	1	97.8	9.9	50	106.7	4.1	7.7
195	Mecarbam	1	0.9919	1–200	1	94.9	6.1	50	100.9	4.9	–4.3
196	Mefenacet	1	0.9965	1–200	1	76.4	12.6	50	96.8	4.6	–0.9
197	Mepanipyrim	1	0.9997	1–200	1	77.8	16.2	50	81.9	7.3	0.3
198	Mephosfolan	1	0.9992	1–200	1	96.1	9.8	50	96.1	3.6	6.5
199	Mepronil	1	0.9914	1–200	1	85.7	9.6	50	99.6	2.1	3.2
200	Metaflumizone	5	0.9991	5–200	5	90.2	11.6	50	93.6	3.4	–1.7
201	Metalaxyl	1	0.9998	1–200	1	97.2	9.0	50	97.0	5.4	0.2
202	Metamifop	1	0.9995	1–200	1	115.5	19.0	50	98.3	5.0	–3.4
203	Metamitron	5	0.9994	5–200	5	84.2	13.2	50	95.5	4.7	–14.1
204	Metazosulfuron	10	0.9985	10–200	10	88.2	5.3	50	91.8	5.4	0.1
205	Metconazole	1	0.9993	1–200	1	103.7	8.8	50	86.5	5.5	–9.2
206	Methabenzthiazuron	1	0.9996	1–200	1	87.0	11.9	50	89.4	4.3	1.9
207	Methamidophos	1	0.9977	1–200	1	58.6	17.1	50	62.8	6.2	13.8
208	Methiocarb	5	0.9939	5–200	5	81.9	13.0	50	92.8	4.6	24.6
209	Methiocarb sulfone	2.5	0.9985	2.5–200	2.5	92.4	13.3	50	97.1	7.5	15.8
210	Methiocarb sulfoxide	1	0.9973	1–200	1	79.9	7.1	50	90.9	6.2	5.5
211	Methomyl	1	0.9971	1–25	1	102.1	15.8	10	91.1	6.3	11.8
212	Methoprotryne	2.5	0.9964	2.5–200	2.5	73.1	7.3	50	81.2	3.0	–3.0
213	Methoxyfenozide	1	0.9981	1–200	1	85.6	19.1	50	96.2	8.1	–1.1
214	Metobromuron	1	0.9999	1–200	1	98.3	4.8	50	91.9	3.4	3.3
215	Metolcarb	5	0.9996	5–200	5	77.4	17.7	50	93.0	7.5	7.9
216	Metominostrboin (Z)	1	0.9989	1–200	1	94.1	8.6	50	100.7	2.4	–0.6
217	Metominostrobin (E)	1	0.9985	1–200	1	92.9	9.8	50	99.4	3.8	1.8
218	Metrafenon	1	0.9999	1–200	1	89.1	17.9	50	90.3	4.5	2.5
219	Mevinphos	5	0.9979	5–200	5	33.9	19.3	50	56.7	12.4	1.8
220	Monocrotophos	5	0.9989	5–200	5	49.7	13.5	50	72.5	9.3	–4.9
221	Monolinuron	1	0.9991	1–200	1	112.6	3.4	50	93.0	3.0	2.3
222	Myclobutanil	2.5	0.9979	2.5–200	2.5	109.7	16.3	50	96.8	4.7	–5.4
223	Napropamide	1	0.9991	1–200	1	72.8	7.8	50	98.9	5.5	–7.6
224	Neburon	2.5	0.9960	2.5–200	2.5	89.3	16.5	50	93.7	5.3	–2.0
225	Nicosulfuron	2.5	0.9996	2.5–200	2.5	89.9	7.3	50	77.5	5.4	25.4
226	Nitenpyram	10	0.9996	10–200	10	76.6	10.9	50	86.6	2.8	–16.1
227	Norflurazon	1	0.9980	1–200	1	97.9	6.8	50	103.4	2.6	–1.6
228	Noruron (Norea)	2.5	0.9990	2.5–200	2.5	81.2	10.3	50	84.6	5.2	2.7
229	Novaluron	5	0.9980	5–200	5	95.7	13.6	50	100.8	5.0	0.5
230	Nuarimol	2.5	0.9997	2.5–200	2.5	71.8	16.8	50	86.8	4.6	8.5
231	Ofurace	1	0.9997	1–200	1	88.9	5.1	50	92.9	4.6	11.0
232	Omethoate	1	0.9997	1–200	1	77.2	5.9	50	89.6	3.7	–10.4
233	Oxadiazon	2.5	0.9984	2.5–200	2.5	116.7	14.0	50	87.7	5.7	–0.4
234	Oxadixyl	1	0.9993	1–200	1	100.9	14.4	50	99.0	5.1	6.5
235	Oxamyl	1	0.9984	1–200	1	89.3	12.1	50	96.2	2.6	4.1
236	Oxaziclomefone	1	0.9998	1–200	1	85.4	10.6	50	89.3	3.6	–7.0
237	Paclobutrazole	2.5	0.9983	2.5–200	2.5	110.4	17.5	50	97.8	2.9	–11.1
238	Penconazole	2.5	0.9996	2.5–200	2.5	73.7	13.2	50	89.2	3.3	–7.4
239	Pencycuron	1	0.9975	1–200	1	78.6	15.9	50	93.2	2.7	–0.3
240	Penoxsulam	2.5	0.9982	2.5–200	2.5	84.9	4.1	50	98.3	4.0	0.2
241	Pentoxaone	10	0.9933	10–200	10	76.2	13.4	50	78.4	4.8	1.1
242	Phenthoate	2.5	0.9976	2.5–200	2.5	99.9	11.6	50	98.3	6.2	–0.2
243	Phosalone	2.5	0.9973	2.5–200	2.5	86.0	10.2	50	89.6	6.0	2.9
244	Phosfolan	1	0.9995	1–200	1	84.9	17.3	50	92.6	6.0	6.1
245	Phosphamidon	1	0.9997	1–200	1	104.4	6.9	50	90.5	7.3	–1.9
246	Phoxim	1	0.9978	1–200	1	83.7	19.4	50	94.5	3.4	–10.3
247	Picolinafen	1	0.9997	1–200	1	80.0	19.5	50	84.2	1.6	–33.5
248	Picoxystrobin	2.5	0.9995	2.5–200	2.5	102.0	7.6	50	102.6	4.9	–12.6
249	Piperonyl butoxide	1	0.9995	1–200	1	90.3	14.4	50	93.7	6.4	–11.9
250	Piperophos	1	0.9992	1–200	1	92.2	8.9	50	96.2	6.3	–1.4
251	Pirimicarb	1	0.9968	1–200	1	71.2	7.0	50	77.2	3.2	3.3
252	Pirimicarb-desmethyl	5	0.9996	5–200	5	68.1	11.4	50	80.1	10.6	0.4
253	Pirimiphos-ethyl	1	0.9971	1–200	1	86.1	12.1	50	89.4	5.0	–17.9
254	Pirimiphos-methyl	1	0.9989	1–200	1	89.7	8.5	50	87.2	2.9	–4.0
255	Probenazole	10	0.9962	10–200	10	101.6	10.4	50	103.0	4.8	3.9
256	Prochloraz	10	0.9996	10–200	10	74.8	5.5	50	78.5	2.1	–2.0
257	Profenofos	2.5	0.9994	2.5–200	2.5	75.6	5.1	50	88.3	2.5	–2.0
258	Promecarb	1	0.9993	1–200	1	90.9	12.2	50	92.7	5.1	1.7
259	Prometryn	1	0.9969	1–200	1	68.7	4.5	50	74.2	7.7	0.5
260	Pronamide (Propyzamide)	2.5	0.9977	2.5–200	2.5	98.1	13.0	50	95.1	3.4	4.5
261	Propachlor	1	0.9996	1–200	1	81.9	8.4	50	84.4	2.6	–0.3
262	Propamocarb	1	0.9996	1–200	1	83.2	6.0	50	91.5	2.6	–3.8
263	Propanil	5	0.9987	5–200	5	91.3	8.5	50	87.3	3.4	–8.4
264	Propaquizafop	5	0.9996	5–200	5	85.2	7.8	50	93.1	1.2	–19.8
265	Propargite	1	1.0000	1–200	1	88.6	3.9	50	90.0	1.9	–9.9
266	Propazine	1	0.9993	1–200	1	86.1	16.4	50	79.9	9.0	–14.8
267	Propiconazole	2.5	0.9995	2.5–200	2.5	92.1	19.6	50	89.6	6.1	–5.3
268	Propoxur	1	0.9943	1–200	1	91.1	11.7	50	99.9	7.7	–4.5
269	Proqunazid	1	0.9998	1–200	1	55.3	3.0	50	57.1	1.1	–5.8
270	Prosulfocarb	1	0.9990	1–200	1	89.8	13.0	50	81.1	2.6	13.2
271	Prothioconazole-desthio	5	0.9992	5–200	5	86.7	14.8	50	84.4	5.4	−18.4
272	Pydiflumetofen	2.5	0.9984	2.5–200	2.5	93.4	9.7	50	93.8	4.4	−3.6
273	Pyracarbolid	1	0.9935	1–200	1	86.6	5.4	50	100.6	4.8	0.8
274	Pyraclofos	1	0.9993	1–200	1	85.8	8.7	50	97.8	4.5	9.3
275	Pyraclonil	1	0.9972	1–200	1	98.3	6.8	50	96.2	6.0	7.7
276	Pyraclostrobin	2.5	0.9988	2.5–200	2.5	103.8	14.7	50	97.4	3.6	−15.3
277	Pyraflufen-ethyl	1	0.9974	1–200	1	78.4	16.7	50	93.6	6.7	7.3
278	Pyraziflumid	1	0.9990	1–200	1	115.8	13.9	50	97.3	5.4	9.6
279	Pyrazolate	5	0.9991	5–200	5	95.9	8.5	50	95.2	3.2	2.2
280	Pyrazophos	1	0.9972	1–200	1	87.5	7.7	50	96.4	3.1	31.6
281	Pyribenzoxim	2.5	0.9900	2.5–200	2.5	103.9	19.0	50	97.4	14.3	−16.0
282	Pyributicarb	1	0.9993	1–200	1	87.5	4.3	50	84.1	4.4	−14.1
283	Pyridaben	1	0.9996	1–200	1	74.9	4.0	50	77.9	1.9	1.0
284	Pyridalyl	10	0.9997	10–200	10	55.3	15.6	50	58.3	3.0	−17.2
285	Pyridaphenthion	1	0.9993	1–200	1	91.5	8.2	50	101.9	11.6	−0.6
286	Pyridate	2.5	0.9964	2.5–200	2.5	105.4	3.7	50	73.9	1.2	4.5
287	Pyrifluquinazon	2.5	0.9968	2.5–200	2.5	75.3	8.0	50	87.6	3.1	−4.6
288	Pyriftalid	1	0.9976	1–200	1	86.2	12.0	50	97.2	6.4	−4.0
289	Pyrimethanil	2.5	0.9993	2.5–200	2.5	64.6	6.7	50	58.9	9.5	−1.8
290	Pyrimidifen	1	0.9993	1–200	1	54.6	16.4	50	67.0	3.1	−46.0
291	Pyriminobac (E)	1	0.9975	1–200	1	100.2	8.9	50	112.6	5.2	−7.9
292	Pyriminobac (Z)	1	0.9939	1–200	1	90.4	5.1	50	106.9	6.7	−4.9
293	Pyrimisulfan	1	0.9998	1–200	1	95.1	11.8	50	95.2	6.0	7.4
294	Pyriproxyfen	1	0.9994	1–200	1	79.2	7.0	50	82.8	5.4	−6.2
295	Pyroquilon	1	0.9994	1–200	1	79.2	19.7	50	91.3	5.4	2.6
296	Quinalphos	5	0.9964	5–200	5	87.5	11.8	50	89.8	2.0	2.0
297	Quinoclamine	10	0.9979	10–200	10	74.1	13.1	50	84.5	5.7	−4.1
298	Quizalofop-ethyl	1	0.9985	1–200	1	70.6	15.1	50	88.3	3.0	−3.4
299	Saflufenacil	2.5	0.9980	2.5–200	2.5	100.9	12.7	50	99.9	6.2	13.2
300	Sethoxydim A	2.5	0.9998	2.5–200	2.5	77.8	3.2	50	80.5	2.2	−3.3
301	Simazine	1	0.9993	1–200	1	87.4	17.9	50	90.0	2.9	1.6
302	Simeconazole	2.5	0.9988	2.5–200	2.5	108.9	10.3	50	95.8	4.5	−2.8
303	Simetryn	1	0.9998	1–200	1	75.6	9.7	50	74.2	4.5	−5.4
304	Spinetoram (J)	1	0.9986	1–200	1	87.9	18.7	50	101.7	17.8	−10.6
305	Spinetoram (L)	1	0.9993	1–200	1	90.4	11.5	50	102.0	4.0	−7.3
306	Spinosyn A	1	0.9978	1–200	1	96.1	14.4	50	87.4	4.1	−6.6
307	Spinosyn D	5	0.9995	5–200	5	108.7	10.3	50	98.8	3.8	−17.0
308	Spirodiclofen	2.5	0.9998	2.5–200	2.5	91.3	4.0	50	85.1	1.6	6.9
309	Spirotetramat	2.5	0.9985	2.5–200	2.5	80.8	13.1	50	101.0	7.6	4.7
310	Spirotetramat-enol	1	0.9988	1–200	1	92.2	10.0	50	90.2	4.1	1.5
311	Spirotetramat-enol-glucoside	10	0.9933	10–200	10	112.2	6.3	50	69.2	4.7	−11.6
312	Spirotetramat-ketohydroxy	10	0.9997	10–200	10	92.1	7.8	50	90.5	2.9	6.0
313	Spirotetramat-monohydroxy	2.5	0.9978	2.5–200	2.5	78.5	17.5	50	96.6	6.3	−4.3
314	Spiroxamine	1	0.9986	1–200	1	82.5	7.2	50	91.5	4.9	0.6
315	Sulfentrazone	2.5	0.9951	2.5–200	2.5	105.8	9.7	50	106.7	4.8	4.7
316	Sulfotep	1	0.9982	1–200	1	87.4	17.3	50	95.6	3.2	3.0
317	Sulfoxaflor	1	0.9976	1–200	1	91.8	3.5	50	99.5	2.5	37.1
318	Sulprofos	2.5	0.9999	2.5–200	2.5	86.6	12.4	50	83.3	1.1	3.7
319	TCMTB	10	0.9993	10–200	10	15.8	18.0	50	16.1	4.9	−6.7
320	Tebuconzole	2.5	0.9989	2.5–200	2.5	90.9	10.4	50	88.6	6.1	13.9
321	Tebufenozide	2.5	0.9912	2.5–200	2.5	97.2	10.6	50	103.7	15.1	−15.8
322	Tebufenpyrad	2.5	0.9997	2.5–200	2.5	88.3	11.1	50	82.4	2.9	−26.6
323	Tebuthiuron	1	0.9990	1–200	1	90.9	14.9	50	89.3	7.3	−2.9
324	Teflubenzuron	1	0.9998	1–200	1	91.3	13.7	50	85.0	4.3	77.3
325	Tepraloxydim	2.5	0.9967	2.5–200	2.5	98.6	19.1	50	98.0	6.7	−1.7
326	Terbuthylazine	2.5	0.9996	2.5–200	2.5	77.5	3.5	50	79.0	5.1	−2.4
327	Terbutryn	10	0.9938	10–200	10	65.9	3.2	50	72.8	2.4	1.3
328	Tetrachlorvinphos	10	0.9999	10–200	10	48.2	7.9	50	70.1	8.6	2.6
329	Tetraconazole	1	0.9996	1–200	1	112.7	17.3	50	97.1	2.6	−7.3
330	Thenylchlor	2.5	0.9990	2.5–200	2.5	89.9	9.2	50	97.4	3.8	−3.8
331	Thiabendazole	1	0.9993	1–200	1	69.0	10.4	50	67.0	3.9	8.2
332	Thiacloprid	1	0.9976	1–200	1	89.7	13.5	50	102.6	3.6	9.7
333	Thiamethoxam	2.5	0.9991	2.5–200	2.5	71.2	17.7	50	97.0	2.1	−5.0
334	Thiazopyr	2.5	0.9982	2.5–200	2.5	89.3	11.4	50	98.9	5.7	0.8
335	Thidiazuron	1	0.9995	1–200	1	76.9	5.2	50	79.8	5.3	14.9
336	Thifensulfuron-methyl	1	0.9998	1–200	1	77.9	13.6	50	82.8	1.5	12.0
337	Thifluzamide	10	0.9948	10–200	10	99.2	16.7	50	105.8	8.1	9.8
338	Thiobencarb	1	0.9997	1–200	1	96.3	15.4	50	80.0	4.6	5.6
339	Thionazin	1	0.9993	1–200	1	108.1	11.1	50	97.7	3.2	1.8
340	Tiadinil	1	0.9994	1–200	1	91.2	5.4	50	84.1	2.8	16.8
341	Tolfenpyrad	2.5	0.9996	2.5–200	2.5	83.7	14.0	50	84.5	5.5	−13.0
342	Triadimefon	5	0.9983	5–200	5	88.6	14.7	50	97.3	4.0	3.0
343	Triazophos	1	0.9947	1–200	1	99.9	11.7	50	116.8	3.6	−7.4
344	Tricyclazole	1	0.9973	1–200	1	79.4	9.1	50	84.5	6.8	−0.3
345	Trifloxystrobin	1	0.9989	1–200	1	118.6	11.1	50	102.7	3.3	−12.2
346	Triflumizole	1	0.9994	1–200	1	89.2	12.4	50	81.5	6.4	−13.5
347	Triflumuron	2.5	0.9998	2.5–200	2.5	96.1	9.8	50	94.2	2.9	−8.6
348	Trimethacarb	1	0.9998	1–200	1	98.3	7.4	50	91.2	2.1	5.1
349	Triticonazole	2.5	0.9995	2.5–200	2.5	75.7	17.3	50	92.6	2.2	−6.3
350	Uniconazole	2.5	0.9990	2.5–200	2.5	82.8	9.7	50	96.7	8.1	−19.3
351	Vamidothion	1	0.9987	1–200	1	82.2	8.9	50	95.0	7.5	−1.1
352	XMC	1	0.9992	1–200	1	90.4	14.1	50	93.2	2.6	13.1
353	Zoxamide	1	0.9989	1–200	1	92.6	19.1	50	100.7	7.1	−2.8

^1^ Concentration of treatment. ^2^ Matrix effect.

**Table 4:** Summary of method validation parameters; LOQ, linearity of calibration (*r^2^*), recovery, and matrix effect for the 353 target pesticides.

Range	No. of Pesticides
**LOQ**	
1 μg/kg	187 (53.0%)
2.5 μg/kg	90 (25.5%)
5 μg/kg	37 (10.5%)
10 μg/kg	39 (11.0%)
Sum	353 (100%)
r^2^	
>0.990 at linear range; LOQ to 200 μg/kg	350 (99.2%)
>0.990 at linear range; LOQ to 25 μg/kg	3 (0.8%)
Sum	353 (100%)
**Recovery**	
Low (RSD 2.3%–19.9%)	
15% to 30%	1 (0.3%)
30% to 70%	30 (8.5%)
70% to 120%	322 (91.2%)
>120%	0 (0.0%)
Sum	353 (100%)
High (RSD 0.8%–17.8%)	
15% to 30%	1 (0.3%)
30% to 70%	19 (5.4%)
70% to 120%	333 (94.3%)
>120%	0 (0.0%)
Sum	353 (100%)
**Matrix Effect**	
<−50% (Strong)	0 (0.0%)
−50% to −20% (Medium)	12 (3.4%)
−20% to 0% (Soft)	164 (46.5%)
0% to 20% (Soft)	169 (47.9%)
20% to 50% (Medium)	6 (1.7%)
>50% (Strong)	2 (0.6%)
Sum	353 (100%)

**Table 5:** Quantitative application results in mealworm samples obtained from commercial mealworm farms and pesticide maximum residue levels (MRLs) in terrestrial invertebrate animals including insects.

Compound Name	Sample no. (μg/kg)				MRL [13] (μg/kg)
	#3	#6	#9	#10	
Flufenoxuron	14.4	1.7	-^1^	-	-
Lufenuron	-	220.7	-	-	20
Noruron (Norea)	3.4	-	19.4	21.0	-

^1^ Not detected.

## Supplementary Materials

The following materials are available online, Table S1: Retention times (t_R_) and multiple reaction monitoring (MRM) transition profiles including the *m*/*z* values of precursors and product ions, declustering potential (DP), entrance potential (EP), collision energy (CE), and cell exit potential (CXP) for 353 target compounds; Table S2: Recovery results of representative pesticides that showed a difference in recovery rate depending on number of acetonitrile-hexane partition rounds (*N* = 1, 2, and 3); Table S3: Comparison of four QuEChERS extraction combinations and distribution of recovery results for 353 target pesticides; Table S4: Recovery results of representative pesticides that showed different recovery rate depending on dSPE sorbent combination (PSA + C18, PSA, and C18). Figure S1: Mealworms grown in wheat bran.
