# Supplementary material for: Liquid Chromatography-Tandem Mass Spectrometry for the Simultaneous Analysis of 353 Pesticides in the Edible Insect Tenebrio molitor Larvae (Mealworms)
Source: Molecules. 2020 Dec 11;25(24):5866. doi: 10.3390/molecules25245866 (PMC7764178; doi:10.3390/molecules25245866)
Supplement: Supplementary file 1 [file molecules-25-05866-s001.pdf]

Supplementary Material

Liquid Chromatography-Tandem Mass Spectrometry for the Simultaneous Analysis of 353 Pesticides in the Edible Insect *Tenebrio molitor* Larvae (Mealworms)

Yongho Shin [1], Chang Jo Kim [1], Sujin Baek [1], Leesun Kim [1], Kyeong-Ae Son [1], Hee-Dong Lee [1], Danbi Kim [1], Jeong-Han Kim [2] and Hyun Ho Noh [1,*]

1 Residual Agrochemical Assessment Division, National Institute of Agricultural Sciences, Rural Development Administration, Iseo-myeon, Wanju-gun, Jeollabuk-do 55365, South Korea; yong6103@korea.kr (Y.S.); rlawkdwh1@gmail.com (C.J.K.); bsj920@gmail.com (S.B.); twosuns@korea.kr (L.K.); sky199@korea.kr (K.-A.S.); yihd@korea.kr (H.-D.L.); danbi6334@korea.kr (D.K.)

2 Pesticide Chemistry and Toxicology Laboratory, Department of Agricultural Biotechnology and Research Institute of Agriculture and Life Sciences, Seoul National University, Seoul 08826, South Korea; kjh2404@snu.ac.kr

* Correspondence: noh1983@korea.kr; Tel.: +82-63-238-3225

Contents

Table S1. Retention times (t_R) and multiple reaction monitoring (MRM) transition profiles including the m/z values of precursors and product ions, declustering potential (DP), entrance potential (EP), collision energy (CE), and cell exit potential (CXP) for 353 target compounds……………………………………………………..…….. p. 2

Table S2. Recovery results of representative pesticides that showed a difference in recovery rate depending on number of acetonitrile-hexane partition rounds (*N* = 1, 2, and 3). Each partitioning round was conducted with acetonitrile extraction using QuEChERS original salts (NaCl and $MgSO_4$) and dSPE cleanup using PSA sorbent……………………………………………………………………..…………………………………….. p. 12

Table S3. Comparison of four QuEChERS extraction combinations and distribution of recovery results for 353 target pesticides. Each extraction was conducted with acetonitrile-hexane partitioning (*N* = 3) followed by dSPE cleanup using PSA..……………………………………………………………………………………………….... p. 13

Table S4. Recovery results of representative pesticides that showed different recovery rate depending on dSPE sorbent combination (PSA+C18, PSA, and C18). Each dSPE cleanup was conducted with acetonitrile extraction using EN 15662 salts followed by acetonitrile-hexane partitioning (*N* = 3)…...…………………………………... p. 14

Figure S1. Mealworms grown in wheat bran…………………………………..………………………………….... p. 15

Table S1. Retention times (t_R) and multiple reaction monitoring (MRM) transition profiles including the m/z values of precursors and product ions, declustering potential (DP), entrance potential (EP), collision energy (CE), and cell exit potential (CXP) for 353 target compounds.

No.	Compound Name	t_R	Ionization	Quantitation ion MRM transition profiles						Confirmation ion MRM transition profiles					
				Precursor ion (m/z)	Product ion (m/z)	DP	EP	CE	CXP	Precursor ion (m/z)	Product ion (m/z)	DP	EP	CE	CXP
1	Abamectin	13.67	Positive	890.5	305.3	86	10	31	12	890.5	567.4	76	10	19	12
2	Acetamiprid	4.05	Positive	223.1	125.9	96	10	29	10	223.1	90.1	96	10	43	8
3	Acibenzolar-S-methyl	8.20	Positive	211.0	135.9	90	10	40	10	211.0	91.0	90	10	27	10
4	Alachlor	9.29	Positive	270.2	238.1	90	10	15	10	270.2	148.0	90	10	34	10
5	Aldicarb sulfoxide	3.38	Positive	207.0	131.9	71	10	9	10	207.1	65.0	51	10	21	6
6	Alidcarb sulfone	3.47	Positive	240.0	86.1	66	10	27	4	240.0	148.0	66	10	19	18
7	Allethrin	11.82	Positive	303.2	134.9	90	10	16	10	303.2	91.0	90	10	55	10
8	Ametoctradin	10.72	Positive	276.2	176.2	141	10	49	8	276.2	149.1	141	10	51	14
9	Ametryn	7.28	Positive	228.2	186.2	90	10	26	10	228.2	95.9	90	10	35	10
10	Anilofos	9.92	Positive	368.0	199.1	90	10	20	10	368.0	125.0	90	10	43	10
11	Aramite	11.77	Positive	352.2	191.3	90	10	17	10	352.2	57.2	90	10	35	10
12	Aspon	12.64	Positive	379.0	115.1	90	10	45	10	379.0	211.0	90	10	26	10
13	Atrazine	6.77	Positive	216.1	174.1	90	10	25	10	216.1	103.9	90	10	25	10
14	Azaconazole	7.06	Positive	300.0	158.9	90	10	36	10	300.0	231.0	90	10	24	10
15	Azimsulfuron	6.69	Positive	425.2	182.1	61	10	21	8	425.2	156.2	61	10	43	8
16	Azinphos-ethyl	8.98	Positive	346.0	132.1	81	10	23	8	346.0	77.2	81	10	55	4
17	Azinphos-methyl	7.35	Positive	318.0	132.1	56	10	21	4	318.0	160.2	56	10	13	4
18	Azoxystrobin	7.55	Positive	404.1	372.1	74	10	19	4	404.1	344.1	74	10	27	4
19	Benalaxyl	10.07	Positive	326.3	148.1	90	10	28	10	326.3	91.1	90	10	56	10
20	Bendiocarb	5.55	Positive	224.1	109.1	61	10	23	4	224.1	167.2	61	10	13	4
21	Benodanil	6.55	Positive	324.1	231.0	90	10	32	10	324.1	203.1	90	10	50	10
22	Benoxacor	7.59	Positive	260.1	149.2	90	10	25	10	262.1	149.2	90	10	25	10
23	Bensulfuron-methyl	7.32	Positive	411.1	148.9	86	10	27	4	411.0	119.0	81	10	51	4
24	Bensulide	9.49	Positive	398.0	158.0	141	10	33	12	398.0	218.1	141	10	23	26
25	Benthiavalicarb-isopropyl	8.47	Positive	382.2	180.1	82	10	41	4	382.2	116.1	82	10	32	4
26	Benzobicyclon	8.24	Positive	447.0	257.1	136	10	33	10	447.0	229.1	136	10	53	18
27	Benzoximate	10.50	Positive	364.0	199.1	56	10	17	4	364.0	105.1	56	10	31	4
28	Benzoylprop-ethyl	10.16	Positive	366.2	104.9	90	10	22	10	368.2	105.1	90	10	22	10
29	Bitertanol	10.43	Positive	338.2	269.2	31	10	13	18	338.2	70.1	31	10	13	6
30	Bixafen	9.57	Positive	413.9	265.9	141	10	31	22	413.9	374.1	141	10	31	32
31	Boscalid	8.12	Positive	343.0	307.0	94	10	27	4	343.0	140.0	116	10	27	4
32	Broflanilide	10.66	Positive	665.0	556.0	201	10	67	14	665.0	506.1	201	10	81	32
33	Broflanilide_DM-8007	11.03	Positive	649.0	242.1	271	10	29	18	649.0	77.2	271	10	129	6

No.	Compound Name	t_R	Ionization	Quantitation ion MRM transition profiles						Confirmation ion MRM transition profiles					
				Precursor ion (m/z)	Product ion (m/z)	DP	EP	CE	CXP	Precursor ion (m/z)	Product ion (m/z)	DP	EP	CE	CXP
34	Broflanilide_S (PFP-OH)-8007	9.13	Positive	661.0	551.0	216	10	75	28	661.0	454.1	216	10	77	8
35	Bromacil	5.66	Positive	261.0	205.0	61	10	19	4	261.0	188.0	61	10	37	4
36	Bromobutide	9.16	Positive	312.2	194.0	90	10	16	10	314.2	196.0	90	10	16	10
37-1	Bromuconazole_1	8.73	Positive	378.1	158.9	91	10	37	12	378.1	70.1	91	10	25	6
37-2	Bromuconazole_2	9.71	Positive	378.1	158.9	91	10	37	12	378.1	70.1	91	10	25	6
38	Bupirimate	9.15	Positive	317.2	166.2	90	10	32	10	317.2	108.0	90	10	35	10
39	Buprofezin	11.68	Positive	306.2	201.1	66	10	17	4	306.2	116.2	66	10	21	4
40	Butocarboxim	4.81	Positive	213.0	75.1	56	10	19	6	213.0	156.1	56	10	15	10
41	Cadusafos	10.83	Positive	271.1	159.0	86	10	19	4	271.0	131.0	71	10	31	4
42	Cafenstrole	8.56	Positive	351.1	100.1	76	10	13	10	351.1	72.1	76	10	43	6
43	Carbaryl	5.92	Positive	202.1	144.9	51	10	15	4	202.1	127.1	71	10	39	4
44	Carbendazim	3.78	Positive	192.2	160.2	76	10	27	4	192.2	132.1	76	10	41	4
45	Carbetamide	5.17	Positive	237.2	120.0	66	10	21	10	237.2	72.1	66	10	41	12
46	Carbofuran	5.61	Positive	222.1	123.0	56	10	29	4	222.1	165.2	56	10	17	4
47	Carbofuran-3-hydroxy	4.06	Positive	238.0	163.0	126	10	21	12	238.0	107.0	126	10	41	10
48	Carboxin	5.97	Positive	236.1	142.9	64	10	21	4	236.0	87.0	61	10	33	4
49	Carfentrazone-ethyl	9.78	Positive	412.0	366.1	85	10	25	4	412.0	346.1	85	10	27	4
50	Carpropamid	9.99	Positive	334.1	103.0	111	10	59	8	334.1	196.1	111	10	19	14
51	Chlorantraniliprole	7.29	Positive	484.0	452.9	51	10	31	36	484.0	285.8	51	10	21	18
52	Chlorbenzuron	9.84	Positive	309.2	156.0	126	10	19	12	309.2	138.9	126	10	39	10
53	Chlorfenvinphos	10.13	Positive	359.0	155.1	90	10	17	10	359.0	99.1	90	10	44	10
54	Chlorimuron-ethyl	8.17	Positive	415.0	185.9	126	10	25	14	415.0	120.9	126	10	59	8
55	Chlorotoluron	6.48	Positive	213.0	72.0	136	10	25	4	213.0	140.0	136	10	33	14
56	Chloroxuron	8.71	Positive	291.2	72.2	90	10	25	10	291.2	164.0	90	10	24	10
57	Chlorpyrifos	12.26	Positive	349.9	96.9	61	10	41	4	350.0	198.0	76	10	25	4
58	Chlorsulfuron	5.77	Positive	358.0	141.0	71	10	23	4	358.0	167.0	91	10	25	4
59	Chromafenozide	8.82	Positive	395.2	175.0	91	10	25	14	395.3	147.1	71	10	59	14
60	Clethodim	11.27	Positive	360.0	164.0	81	10	25	4	360.1	268.2	84	10	17	4
61	Clofentezine	10.58	Positive	303.0	138.0	96	10	21	4	303.1	102.1	76	10	47	4
62	Clomazone	7.57	Positive	240.1	125.0	66	10	27	4	240.0	89.0	66	10	65	4
63	Clothianidin	3.92	Positive	250.0	132.0	71	10	21	4	250.0	169.1	94	10	19	4
64	Coumaphos	10.05	Positive	363.1	227.1	90	10	36	10	363.1	307.1	90	10	25	10
65	Crotoxyphos	8.06	Positive	332.3	211.0	90	10	11	10	332.3	127.0	90	10	34	10
66	Crufomate	9.48	Positive	292.0	235.9	136	10	27	18	292.0	107.9	136	10	37	10
67	Cyanazine	5.12	Positive	241.2	214.1	90	10	23	10	243.2	216.1	90	10	23	10
68	Cyazofamid	9.14	Positive	325.0	108.0	76	10	19	4	325.0	261.0	76	10	15	4
69	Cyclosulfamuron	8.72	Positive	422.0	261.0	56	10	25	24	422.0	218.0	56	10	37	16

No.	Compound Name	t_R	Ionization	Quantitation ion MRM transition profiles						Confirmation ion MRM transition profiles					
				Precursor ion (m/z)	Product ion (m/z)	DP	EP	CE	CXP	Precursor ion (m/z)	Product ion (m/z)	DP	EP	CE	CXP
70	Cyflufenamid	10.36	Positive	413.1	295.2	24	10	21	4	413.1	241.0	24	10	32	4
71	Cyhalofop-butyl	10.88	Positive	375.1	256.1	76	10	21	4	375.2	120.1	76	10	41	4
72	Cymoxanil	4.42	Positive	199.0	128.2	76	10	13	6	199.0	111.1	76	10	25	4
73	Cyprazine	6.88	Positive	228.1	186.1	90	10	25	10	230.1	188.1	90	10	25	10
74	Cyprodinil	9.74	Positive	226.1	93.1	90	10	50	10	226.1	77.0	90	10	60	10
75	Daimuron	8.50	Positive	269.1	151.1	56	10	17	12	269.1	119.1	56	10	27	10
76	Demeton-S	7.64	Positive	259.0	89.1	46	10	19	6	259.0	61.0	46	10	45	8
77	Demeton-S-methyl	5.75	Positive	231.0	89.0	56	10	13	8	231.0	61.0	56	10	41	8
78	Demeton-S-methyl sulfone	3.59	Positive	262.9	169.0	101	10	21	18	262.9	109.0	101	10	41	10
79	Demeton-S-methyl sulfoxide	3.52	Positive	247.0	169.0	61	10	19	12	247.0	109.0	61	10	37	8
80	Desmetryn	6.14	Positive	214.1	172.1	90	10	25	10	214.1	82.2	90	10	40	10
81	Diazinon	10.21	Positive	305.1	169.1	76	10	27	4	305.1	153.3	76	10	27	4
82	Diclobutrazol	9.77	Positive	328.1	70.1	90	10	50	10	330.1	70.1	90	10	50	10
83	Dicrotophos	3.70	Positive	238.0	112.1	81	10	15	10	238.0	127.0	81	10	21	12
84	Diethatyl-ethyl	9.67	Positive	312.3	162.2	90	10	35	10	312.3	147.1	90	10	52	10
85	Diethofencarb	7.79	Positive	268.1	226.1	69	10	15	4	268.0	180.0	66	10	23	4
86	Difenoconazole	10.71	Positive	406.1	251.0	90	10	35	10	406.1	337.0	90	10	25	10
87	Diflubenzuron	9.42	Positive	311.0	158.0	66	10	19	4	311.0	141.2	66	10	47	4
88	Diflufenican	10.95	Positive	395.1	266.1	90	10	33	10	395.1	246.2	90	10	50	10
89	Dimepiperate	11.10	Positive	264.1	145.9	54	10	15	4	264.1	119.0	54	10	23	4
90	Dimethachlor	7.29	Positive	256.2	224.2	90	10	20	10	256.2	148.2	90	10	35	10
91	Dimethametryn	9.38	Positive	256.2	186.1	71	10	27	4	256.2	68.0	71	10	61	4
92	Dimethenamide	8.14	Positive	276.1	244.1	66	10	17	4	276.1	168.3	66	10	33	4
93	Dimethoate	4.17	Positive	230.0	198.9	90	10	12	10	230.0	125.1	90	10	29	10
94-1	Dimethomorph_1	7.83	Positive	388.1	301.1	81	10	27	4	388.1	165.2	86	10	45	4
94-2	Dimethomorph_2	8.28	Positive	388.1	301.1	81	10	27	4	388.1	165.2	86	10	45	4
95	Dimethylaminosulfotoluidide (DMST)	5.72	Positive	215.2	106.1	90	10	20	10	215.2	77.1	90	10	55	10
96	Dimethylvinphos (E)	9.13	Positive	331.1	127.0	90	10	16	10	333.1	127.0	90	10	16	10
97	Dimethylvinphos (Z)	8.71	Positive	331.1	127.0	90	10	15	10	333.1	127.0	90	10	15	10
98	Diniconazole	10.67	Positive	326.0	70.0	74	10	45	4	326.0	159.0	76	10	43	4
99	Dinotefuran	3.40	Positive	203.2	129.1	71	10	17	8	203.2	114.1	71	10	17	10
100	Diphenamid	7.24	Positive	240.1	134.1	89	10	29	4	240.2	167.2	41	7.5	14	33
101	Dithiopyr	11.06	Positive	402.1	353.9	91	10	23	4	402.1	272.1	91	10	38	4
102	Diuron	7.01	Positive	233.1	72.1	101	10	21	6	233.1	160.0	101	10	37	12
103	Edifenphos	9.90	Positive	310.9	109.0	131	10	47	10	310.9	111.1	131	10	29	6
104	Esprocarb	11.77	Positive	266.1	91.0	76	10	35	4	266.1	65.0	76	10	79	4
105	Etaconazole	9.13	Positive	328.1	159.1	90	10	36	10	330.1	161.1	90	10	36	10

No.	Compound Name	tR	Ionization	Quantitation ion MRM transition profiles						Confirmation ion MRM transition profiles					
				Precursor ion (m/z)	Product ion (m/z)	DP	EP	CE	CXP	Precursor ion (m/z)	Product ion (m/z)	DP	EP	CE	CXP
106	Ethaboxam	6.23	Positive	321.0	183.1	86	10	33	4	321.0	200.1	86	10	39	4
107	Ethiofencarb	6.26	Positive	226.1	169.0	61	10	9	12	226.1	164.0	61	10	11	16
108	Ethoprophos	9.17	Positive	243.0	97.0	71	10	41	4	243.0	131.0	66	10	29	4
109	Ethoxysulfuron	8.41	Positive	399.1	261.0	59	10	21	4	399.1	218.2	41	7	18	33
110	Etofenprox	14.54	Positive	394.2	177.1	61	10	21	18	394.2	107.0	61	10	61	12
111	Etoxazole	12.40	Positive	360.2	141.0	76	10	37	4	360.1	57.2	76	10	45	4
112	Etrimfos	10.08	Positive	293.1	125.0	64	10	33	4	293.0	265.0	61	10	21	4
113	Famoxadone	10.08	Positive	392.0	331.0	51	10	15	4	392.2	238.0	54	10	23	4
114	Fenamiphos	9.45	Positive	304.2	217.1	81	10	31	4	304.2	202.1	81	10	47	4
115	Fenamiphos sulfone	5.60	Positive	337.1	267.1	156	10	29	20	337.1	109.1	156	10	47	10
116	Fenamiphos sulfoxide	5.42	Positive	320.0	233.1	156	10	33	16	320.0	171.0	156	10	29	14
117	Fenazaquin	13.43	Positive	307.2	161.2	76	10	31	4	307.0	147.0	61	10	25	4
118	Fenbuconazole	9.24	Positive	337.1	125.1	81	10	37	4	337.1	70.0	81	10	35	4
119	Fenfuram	6.10	Positive	202.1	109.0	90	10	27	10	202.1	120.1	90	10	21	10
120	Fenhexamid	8.95	Positive	302.1	97.2	101	10	33	4	302.0	55.0	101	10	57	4
121	Fenobucarb	7.74	Positive	208.1	95.0	71	10	19	6	208.1	152.0	71	10	11	10
122	Fenothiocarb	9.65	Positive	254.1	72.0	90	10	21	10	254.1	160.1	90	10	14	10
123	Fenoxanil	9.46	Positive	329.1	302.1	90	10	16	10	331.1	304.1	90	10	16	10
124	Fenoxaprop-ethyl	11.41	Positive	362.1	288.1	206	10	25	18	362.1	77.1	206	10	77	8
125	Fenoxycarb	9.54	Positive	302.1	88.0	84	10	29	4	302.2	116.2	68	10	16	4
126	Fenpropathrin	12.59	Positive	367.2	125.2	90	10	21	10	367.2	350.1	90	10	11	10
127	Fenpropimorph	7.45	Positive	304.2	147.2	111	10	39	8	304.2	117.2	111	10	79	10
128	Fenpyroximate	12.74	Positive	422.2	366.2	91	10	21	24	422.2	138.0	91	10	41	10
129	Fenquinotrione	7.35	Positive	425.1	285.0	156	10	37	12	425.1	179.1	156	10	91	14
130	Fenthion	10.06	Positive	279.0	169.1	136	10	27	12	279.0	246.9	136	10	17	22
131	Fenthion oxon	7.35	Positive	263.0	231.0	131	10	23	10	263.0	216.0	131	10	31	14
132	Fenthion oxon sulfoxide	3.92	Positive	279.0	104.0	151	10	37	10	279.0	109.1	151	10	53	12
133	Fenthion sulfoxide	5.65	Positive	295.1	280.0	141	10	27	18	295.1	109.1	121	10	41	10
134	Fenthion-sulfone	5.90	Positive	310.9	124.9	126	10	25	10	310.9	278.9	126	10	25	10
135	Fentrazamide	9.91	Positive	350.2	154.2	61	10	16	4	350.2	197.3	61	10	11	4
136	Ferimzone	7.62	Positive	255.1	91.1	121	10	45	8	255.1	65.1	121	10	73	6
137	Fipronil	9.41	Negative	434.8	329.9	-95	-10	-24	-21	434.8	249.9	-95	-10	-38	-19
138	Fipronil-sulfone	9.88	Negative	450.8	415.0	-115	-10	-26	-29	450.8	282.0	-115	-10	-40	-11
139	Flamprop-isopropyl	10.00	Positive	364.1	105.1	90	10	28	10	364.1	77.1	90	10	75	10
140	Flonicamid	3.65	Positive	230.0	203.2	76	10	25	4	230.0	97.8	76	10	47	4
141	Fluacrypyrim	10.76	Positive	427.2	145.0	55	10	34	4	427.2	205.1	55	10	14	4
142	Fluazinam	11.74	Negative	462.8	415.9	-65	-10	-30	-11	462.8	398.1	-65	-10	-26	-29

No.	Compound Name	tR	Ionization	Quantitation ion MRM transition profiles						Confirmation ion MRM transition profiles					
				Precursor ion (m/z)	Product ion (m/z)	DP	EP	CE	CXP	Precursor ion (m/z)	Product ion (m/z)	DP	EP	CE	CXP
143	Flucetosulfuron	7.62	Positive	488.0	156.1	86	10	25	10	488.0	273.0	86	10	35	22
144	Fludioxonil	7.98	Negative	246.9	180.0	-115	-10	-42	-15	246.9	126.0	-115	-10	-42	-11
145	Flufenacet	9.06	Positive	364.1	194.2	61	10	17	4	364.1	152.2	61	10	25	4
146	Flufenoxuron	12.29	Positive	489.1	158.2	81	10	25	4	489.1	141.2	81	10	65	4
147	Fluometuron	6.31	Positive	233.1	145.1	121	10	47	8	233.1	168.0	81	10	39	12
148	Fluopicolide	8.38	Positive	383.0	172.9	81	10	33	12	383.0	109.0	81	10	87	8
149	Fluopyram	8.78	Positive	397.1	145.0	90	10	75	10	397.1	173.0	90	10	36	10
150	Flupyradifuron	4.04	Positive	289.0	125.9	106	10	39	10	289.0	72.9	106	10	97	20
151	Fluquinconazole	8.87	Positive	376.0	307.1	94	10	33	4	376.0	349.0	91	10	25	4
152	Fluridone	7.36	Positive	330.0	309.1	201	10	49	26	330.0	294.1	201	10	63	22
153	Flurochloridone	8.63	Positive	312.2	292.2	90	10	30	10	312.2	145.1	90	10	65	10
154	Flurtamone	7.74	Positive	334.1	247.0	90	10	37	10	334.1	178.1	90	10	60	10
155	Flusilazole	9.42	Positive	316.1	247.1	76	10	25	4	316.1	165.1	76	10	39	4
156	Fluthiacet-methyl	9.73	Positive	404.1	274.1	90	10	39	10	404.1	239.1	90	10	50	10
157	Flutianil	9.65	Positive	427.0	192.2	90	10	34	10	427.0	411.1	90	10	43	10
158	Flutolanil	8.25	Positive	324.1	262.1	109	10	23	4	324.1	242.2	109	10	31	4
159	Flutriafol	6.61	Positive	302.2	70.1	91	10	21	6	302.2	95.1	91	10	71	8
160	Fluxapyroxad	8.31	Positive	382.0	362.0	186	10	21	20	382.0	342.0	186	10	29	16
161	Forchlorfenuron	6.90	Positive	248.2	129.1	27	10	24	4	248.0	93.1	27	10	48	4
162	Fosthiazate	6.29	Positive	284.0	104.0	101	10	27	4	284.0	228.0	101	10	15	4
163	Halosulfuron-methyl	8.86	Positive	435.1	182.0	76	10	27	14	435.1	139.1	76	10	63	12
164	Haloxyfop	9.69	Negative	359.9	288.0	-80	-10	-22	-21	359.9	195.9	-80	-10	-58	-13
165	Heptenophos	7.01	Positive	251.1	127.0	90	10	18	10	251.1	125.1	90	10	20	10
166	Hexaconazole	10.30	Positive	314.1	70.1	106	10	25	6	314.1	159.0	106	10	43	12
167	Hexazinone	5.64	Positive	253.2	171.1	56	10	21	4	253.2	71.1	86	10	43	4
168	Hexythiazox	12.15	Positive	353.1	228.0	81	10	21	4	353.1	168.1	76	10	37	4
169	Imazalil	6.08	Positive	297.1	159.0	61	10	29	14	297.1	255.0	61	10	25	12
170	Imazamox	4.28	Positive	306.0	246.1	151	10	33	16	306.0	86.2	151	10	35	8
171	Imazapic	4.39	Positive	276.1	163.1	91	10	37	6	276.1	216.3	91	10	33	10
172	Imazaquin	5.47	Positive	312.1	199.1	26	10	37	18	312.1	267.2	26	10	29	32
173	Imazethapyr	5.04	Positive	290.1	245.2	101	10	29	18	290.1	177.0	101	10	37	16
174	Imazosulfuron	8.14	Negative	411.0	230.0	-125	-10	-24	-7	411.0	154.1	-125	-10	-34	-7
175	Imicyafos	4.97	Positive	305.1	201.1	41	4.5	14	33	305.1	235.2	41	4.5	14	23
176	Imidacloprid	3.84	Positive	256.1	209.0	89	10	23	4	256.1	175.0	89	10	25	4
177	Inabenfide	7.85	Negative	336.9	122.0	-125	-10	-22	-11	336.9	230.9	-125	-10	-30	-13
178	Indanofan	9.21	Positive	341.2	187.2	90	10	18	10	341.2	175.0	90	10	16	10
179	Ipconazole	11.06	Positive	334.2	70.2	111	10	53	6	334.2	125.0	111	10	47	10

No.	Compound Name	t_R	Ionization	Quantitation ion MRM transition profiles						Confirmation ion MRM transition profiles					
				Precursor ion (m/z)	Product ion (m/z)	DP	EP	CE	CXP	Precursor ion (m/z)	Product ion (m/z)	DP	EP	CE	CXP
180	Iprobenfos	9.71	Positive	289.1	91.2	31	10	40	4	289.1	205.0	31	10	14	4
181	Iprovalicarb	8.90	Positive	321.3	119.2	71	10	27	6	321.2	203.2	61	10	12	4
182	Isoprocarb	6.70	Positive	194.1	95.1	56	10	21	8	194.1	137.0	56	10	13	8
183	Isoprothiolane	8.37	Positive	291.1	231.1	51	10	17	4	291.1	189.0	21	3.5	12	29
184	Isoproturon	6.89	Positive	207.1	72.1	111	10	25	6	207.1	165.1	111	10	19	8
185	Isopyrazam	10.73	Positive	360.1	244.1	76	10	27	4	360.1	320.1	76	10	25	4
186	Isoxaben	8.19	Positive	333.2	165.2	91	10	27	12	333.2	107.2	91	10	83	10
187	Isoxathion	10.36	Positive	314.2	105.0	90	10	20	10	314.2	97.1	90	10	50	10
188	Kresoxim-methyl	9.85	Positive	314.1	115.9	54	10	21	4	314.0	206.0	51	10	13	4
189	Lenacil	6.81	Positive	235.1	153.1	61	10	21	4	235.1	136.0	61	10	47	16
190	Linuron	7.85	Positive	249.0	159.9	89	10	23	4	249.0	182.1	89	10	21	4
191	Lufenuron	11.66	Positive	510.9	158.2	79	10	27	4	510.9	141.2	79	10	67	4
192	Malaoxon	5.55	Positive	315.0	99.0	96	10	39	8	315.0	127.0	96	10	17	12
193	Malathion	8.38	Positive	331.0	127.0	96	10	17	10	331.0	285.0	96	10	11	6
194	Mandipropamid	8.08	Positive	412.1	328.1	31	10	22	4	412.1	125.1	31	10	60	4
195	Mecarbam	9.07	Positive	330.1	227.0	90	10	12	10	330.1	199.0	90	10	20	10
196	Mefenacet	8.66	Positive	299.1	148.1	24	10	19	4	299.0	120.1	24	10	35	4
197	Mepanipyrim	8.97	Positive	224.1	106.1	156	10	33	8	224.1	66.1	156	10	55	8
198	Mephosfolan	5.33	Positive	269.9	139.9	76	10	37	14	269.9	195.9	76	10	19	14
199	Mepronil	8.43	Positive	270.1	119.0	71	10	31	4	270.2	228.1	71	10	19	4
200-1	Metaflumizone_1	10.74	Positive	507.0	178.0	86	10	29	10	507.0	287.2	86	10	31	10
200-2	Metaflumizone_2	11.45	Positive	507.0	178.0	86	10	29	10	507.0	287.2	86	10	31	10
201	Metalaxyl	6.88	Positive	280.1	220.0	71	10	19	4	280.2	192.3	71	10	21	4
202	Metamifop	11.35	Positive	441.1	288.1	41	10.5	16	25	441.1	180.2	41	10.5	16	27
203	Metamitron	4.23	Positive	203.1	175.2	101	10	23	10	203.1	104.1	101	10	33	4
204	Metazosulfuron	7.79	Positive	476.0	182.0	31	4.5	22	25	476.0	295.2	31	4.5	22	23
205	Metconazole	10.37	Positive	320.1	70.1	76	10	45	4	320.0	125.0	81	10	49	4
206	Methabenzthiazuron	6.73	Positive	222.2	165.1	71	10	23	4	222.1	150.3	71	10	41	4
207	Methamidophos	3.22	Positive	142.1	94.0	71	10	19	8	142.1	112.1	71	10	21	6
208	Methiocarb	7.98	Positive	226.1	169.1	81	10	13	12	226.1	107.0	81	10	51	8
209	Methiocarb sulfone	4.07	Positive	275.0	122.2	36	10	27	8	275.0	201.0	36	10	19	8
210	Methiocarb sulfoxide	3.87	Positive	242.1	185.0	81	10	19	14	242.1	122.2	81	10	39	12
211	Methomyl	3.61	Positive	163.1	88.1	81	10	13	4	163.1	106.0	81	10	13	4
212	Methoprotryne	7.31	Positive	272.2	198.1	90	10	31	10	272.2	240.1	90	10	26	10
213	Methoxyfenozide	8.37	Positive	369.2	149.1	79	10	23	4	369.2	313.2	79	10	13	4
214	Metobromuron	6.62	Positive	259.0	170.0	76	10	25	4	259.0	148.2	76	10	21	4
215	Metolcarb	5.20	Positive	166.1	109.1	41	10	17	10	166.1	91.1	41	10	33	6

				Quantitation ion MRM transition profiles						Confirmation ion MRM transition profiles					
No.	Compound Name	t_R	Ionization	Precursor ion (m/z)	Product ion (m/z)	DP	EP	CE	CXP	Precursor ion (m/z)	Product ion (m/z)	DP	EP	CE	CXP
216	Metominostrboin (Z)	6.70	Positive	285.1	196.1	66	10	19	10	285.1	77.1	66	10	53	8
217	Metominostrobin (E)	7.12	Positive	285.1	194.1	106	10	27	8	285.1	196.1	106	10	21	12
218	Metrafenon	10.60	Positive	409.1	209.1	46	10	19	14	409.1	226.9	46	10	29	14
219-1	Mevinphos_1	4.07	Positive	225.0	127.0	66	10	21	12	225.0	193.0	66	10	11	16
219-2	Mevinphos_2	4.46	Positive	225.0	127.0	66	10	21	12	225.0	193.0	66	10	11	16
220	Monocrotophos	3.63	Positive	224.1	127.0	86	10	21	4	224.0	98.0	86	10	17	4
221	Monolinuron	6.23	Positive	215.1	126.0	90	10	24	10	215.1	99.1	90	10	47	10
222	Myclobutanil	8.49	Positive	289.1	70.1	69	10	33	4	289.1	125.2	69	10	39	4
223	Napropamide	9.16	Positive	272.1	129.3	71	10	21	4	272.0	171.0	71	10	23	4
224	Neburon	9.64	Positive	275.0	88.0	111	10	21	10	275.0	114.1	111	10	21	8
225	Nicosulfuron	5.26	Positive	411.1	182.1	81	10	25	4	411.0	213.0	101	10	23	4
226	Nitenpyram	3.50	Positive	271.0	225.1	86	10	15	6	271.0	126.0	86	10	35	12
227	Norflurazon	7.00	Positive	304.1	284.1	90	10	32	10	304.1	160.0	90	10	40	10
228	Noruron (Norea)	8.04	Positive	223.2	67.1	146	10	47	6	223.2	93.0	146	10	33	8
229	Novaluron	10.94	Negative	490.8	304.9	-145	-10	-22	-27	490.8	85.0	-145	-10	-78	-7
230	Nuarimol	7.82	Positive	315.1	252.1	101	10	31	22	315.1	81.1	101	10	49	8
231	Ofurace	5.54	Positive	282.1	254.0	84	10	17	4	282.0	160.0	86	10	29	4
232	Omethoate	3.32	Positive	214.0	182.9	66	10	15	14	214.0	125.0	66	10	29	10
233	Oxadiazon	11.83	Positive	362.1	303.1	61	10	25	22	362.1	219.9	61	10	33	8
234	Oxadixyl	4.85	Positive	279.2	219.2	66	10	15	4	279.2	132.1	66	10	41	4
235	Oxamyl	3.47	Positive	237.1	72.0	63	10	25	4	237.1	90.1	63	10	11	4
236	Oxaziclomefone	11.37	Positive	376.1	190.1	91	10	19	14	376.1	161.1	91	10	37	10
237	Paclobutrazole	8.31	Positive	294.1	70.1	51	10	23	8	294.1	125.0	51	10	53	10
238	Penconazole	9.86	Positive	284.1	158.9	81	10	39	4	284.1	70.0	81	10	31	4
239	Pencycuron	10.60	Positive	329.1	125.1	71	10	39	10	329.1	89.1	71	10	89	8
240	Penoxsulam	5.85	Positive	484.0	195.0	186	10	37	14	484.0	164.2	186	10	43	12
241	Pentoxaone	11.44	Positive	354.3	286.0	126	10	17	22	354.3	186.0	126	10	37	14
242	Phenthoate	9.71	Positive	321.0	163.1	76	10	17	4	321.0	79.0	76	10	51	4
243	Phosalone	10.46	Positive	368.0	182.1	90	10	21	10	370.0	184.1	90	10	21	10
244	Phosfolan	4.66	Positive	256.0	139.8	86	10	35	12	256.0	227.9	86	10	17	8
245	Phosphamidon	4.93	Positive	300.1	174.0	126	10	19	12	300.1	127.0	126	10	31	10
246	Phoxim	10.32	Positive	299.0	129.1	66	10	17	4	299.0	77.0	66	10	41	4
247	Picolinafen	11.60	Positive	377.1	238.1	90	10	38	10	377.1	145.1	90	10	70	10
248	Picoxystrobin	9.47	Positive	368.1	145.1	90	10	30	10	368.1	205.1	90	10	14	10
249	Piperonyl butoxide	11.92	Positive	356.3	177.1	90	10	17	10	356.3	119.0	90	10	49	10
250	Piperophos	10.88	Positive	354.1	171.1	64	10	29	4	354.1	255.0	64	10	19	4
251	Pirimicarb	5.68	Positive	239.1	72.1	56	10	31	4	239.2	182.2	73	10	21	4

No.	Compound Name	tR	Ionization	Quantitation ion MRM transition profiles						Confirmation ion MRM transition profiles					
				Precursor ion (m/z)	Product ion (m/z)	DP	EP	CE	CXP	Precursor ion (m/z)	Product ion (m/z)	DP	EP	CE	CXP
252	Pirimicarb-desmethyl	4.06	Positive	225.1	72.2	96	10	41	6	225.1	168.2	96	10	19	14
253	Pirimiphos-ethyl	11.83	Positive	334.1	198.1	86	10	31	14	334.1	182.2	86	10	29	14
254	Pirimiphos-methyl	10.56	Positive	306.1	164.1	27	10	29	4	306.1	108.0	27	10	43	4
255	Probenazole	5.09	Positive	224.1	41.0	61	10	21	4	224.1	184.1	61	10	20	4
256	Prochloraz	10.25	Positive	376.1	308.0	90	10	16	10	376.1	266.0	90	10	23	10
257	Profenofos	11.41	Positive	372.9	302.9	86	10	25	4	373.0	97.0	76	10	43	4
258	Promecarb	8.25	Positive	208.1	109.1	81	10	21	4	208.1	151.1	81	10	13	16
259	Prometryn	8.47	Positive	242.1	158.1	90	10	31	10	242.1	200.2	90	10	25	10
260	Pronamide (Propyzamide)	8.49	Positive	256.1	189.9	90	10	20	10	256.1	172.9	90	10	30	10
261	Propachlor	6.89	Positive	212.1	170.1	90	10	20	10	212.1	94.0	90	10	35	10
262	Propamocarb	3.32	Positive	189.2	102.0	51	10	25	10	189.2	144.1	51	10	17	8
263	Propanil	7.94	Positive	218.0	162.0	82	10	21	4	218.0	127.1	82	10	37	4
264	Propaquizafop	11.70	Positive	444.1	100.0	116	10	23	8	444.1	299.0	116	10	31	18
265	Propargite	12.44	Positive	368.2	231.1	51	10	15	16	368.2	175.1	51	10	23	14
266	Propazine	7.92	Positive	230.2	146.0	90	10	31	10	230.2	188.1	90	10	25	10
267	Propiconazole	10.13	Positive	342.2	159.0	90	10	40	10	342.2	69.1	90	10	25	10
268	Propoxur	5.56	Positive	210.1	111.1	58	10	19	4	210.1	168.1	58	10	11	4
269	Proqunazid	13.22	Positive	373.0	331.0	166	10	21	10	373.0	289.1	166	10	33	14
270	Prosulfocarb	11.34	Positive	252.1	91.1	131	10	33	8	252.1	65.1	131	10	75	10
271	Prothioconazole-desthio	9.33	Positive	312.1	70.0	86	10	27	8	312.1	125.0	86	10	47	10
272	Pydiflumetofen	10.66	Positive	426.1	192.9	126	10	49	14	426.1	123.1	126	10	111	10
273	Pyracarbolid	5.81	Positive	218.2	125.1	90	10	25	10	218.2	97.2	90	10	36	10
274	Pyraclofos	10.41	Positive	361.0	257.0	91	10	30	4	361.1	138.1	91	10	49	4
275	Pyraclonil	6.20	Positive	315.1	169.0	141	10	43	4	315.1	241.2	141	10	27	4
276	Pyraclostrobin	10.28	Positive	388.1	194.0	59	10	19	4	388.0	163.0	51	10	29	4
277	Pyraflufen-ethyl	9.94	Positive	413.0	339.1	76	10	25	14	413.0	252.9	76	10	47	10
278	Pyraziflumid	8.86	Positive	380.0	146.9	181	10	41	6	380.0	68.9	181	10	117	32
279	Pyrazolate	10.54	Positive	439.0	173.1	46	9	20	27	439.0	155.2	46	9	20	27
280	Pyrazophos	10.52	Positive	374.1	222.1	81	10	29	4	374.1	194.0	101	10	43	4
281	Pyribenzoxim	11.50	Positive	610.0	413.2	11	5.5	22	23	610.0	180.1	11	5.5	22	39
282	Pyributicarb	12.05	Positive	331.1	181.1	25	10	21	4	331.1	108.0	25	10	40	4
283	Pyridaben	13.28	Positive	365.1	309.1	64	10	19	4	365.0	147.0	61	10	31	4
284	Pyridalyl	15.29	Positive	492.0	109.0	141	10	37	8	492.0	111.1	141	10	37	12
285	Pyridaphenthion	8.55	Positive	341.0	189.0	86	10	29	4	341.0	205.0	26	10	35	4
286	Pyridate	13.88	Positive	379.1	207.0	86	10	23	14	379.1	351.1	86	10	13	52
287	Pyrifluquinazon	8.60	Positive	465.1	423.1	141	10	29	14	465.1	92.2	141	10	69	6
288	Pyriftalid	7.50	Positive	319.1	139.0	161	10	39	10	319.1	179.0	161	10	41	14

No.	Compound Name	t_R	Ionization	Quantitation ion MRM transition profiles						Confirmation ion MRM transition profiles					
				Precursor ion (m/z)	Product ion (m/z)	DP	EP	CE	CXP	Precursor ion (m/z)	Product ion (m/z)	DP	EP	CE	CXP
289	Pyrimethanil	7.83	Positive	200.0	107.0	91	10	33	4	200.0	82.0	91	10	35	4
290	Pyrimidifen	11.74	Positive	378.2	184.1	166	10	31	14	378.2	157.1	166	10	53	14
291	Pyriminobac (E)	8.22	Positive	362.2	330.1	101	10	19	14	362.2	284.1	101	10	39	16
292	Pyriminobac (Z)	7.56	Positive	362.2	330.2	86	10	21	22	362.2	244.0	96	10	37	18
293	Pyrimisulfan	7.07	Positive	420.1	370.1	46	10	25	32	420.1	255.2	46	10	37	4
294	Pyriproxyfen	12.08	Positive	322.1	96.2	54	10	21	4	322.0	185.0	51	10	29	4
295	Pyroquilon	5.44	Positive	174.1	132.1	51	10	31	12	174.1	117.2	51	10	45	10
296	Quinalphos	9.81	Positive	299.0	97.1	36	10	33	4	299.0	163.0	61	10	29	4
297	Quinoclamine	5.27	Positive	208.1	105.0	191	10	33	6	208.1	89.0	191	10	51	6
298	Quizalofop-ethyl	11.49	Positive	373.1	298.9	94	10	25	4	373.0	271.0	116	10	33	4
299	Saflufenacil	7.49	Positive	501.1	197.9	111	10	59	14	501.1	349.1	111	10	35	32
300	Sethoxydim A	11.76	Positive	328.1	178.2	61	10	27	4	328.1	180.2	61	10	33	4
301	Simazine	5.66	Positive	202.2	132.2	90	10	26	10	202.2	103.9	90	10	33	10
302	Simeconazole	9.01	Positive	294.1	70.1	90	10	22	10	294.1	135.1	90	10	29	10
303	Simetryn	6.10	Positive	214.1	124.0	90	10	27	10	214.1	144.0	90	10	27	10
304	Spinetoram (J)	10.58	Positive	748.4	142.1	91	10	39	12	748.4	98.2	91	10	89	6
305	Spinetoram (L)	11.12	Positive	760.4	142.1	71	10	37	12	760.4	98.2	71	10	97	12
306	Spinosyn A	9.95	Positive	732.4	142.1	86	10	37	12	732.4	98.2	86	10	95	6
307	Spinosyn D	10.49	Positive	746.4	142.2	81	10	39	6	746.4	98.2	81	10	97	8
308	Spirodiclofen	12.79	Positive	411.1	313.0	121	10	17	12	411.1	71.2	121	10	27	6
309	Spirotetramat	4.23	Positive	278.3	174.0	131	10	27	22	278.3	154.0	106	10	27	22
310	Spirotetramat-enol	6.24	Positive	302.1	216.2	56	10	37	6	302.1	270.2	56	10	29	10
311	Spirotetramat-enol-glucoside	3.68	Positive	464.1	302.2	76	10	27	14	464.1	270.0	76	10	43	22
312	Spirotetramat-ketohydroxy	7.06	Positive	318.1	214.2	91	10	37	18	318.1	268.2	91	10	27	10
313	Spirotetramat-monohydroxy	5.43	Positive	304.2	254.1	156	10	25	20	304.2	211.0	136	10	31	16
314	Spiroxamine	7.50	Positive	298.2	144.1	90	10	28	10	298.2	100.1	90	10	40	10
315	Sulfentrazone	5.75	Positive	404.0	307.0	86	10	35	12	404.0	272.9	86	10	47	10
316	Sulfotep	9.88	Positive	323.1	115.0	90	10	40	10	323.1	171.1	90	10	20	10
317	Sulfoxaflor	4.23	Negative	275.9	213.0	-75	-10	-22	-15	275.9	212.0	-75	-10	-22	-25
318	Sulprofos	12.32	Positive	323.1	218.9	90	10	23	10	323.1	139.2	90	10	40	10
319	TCMTB	7.88	Positive	239.0	180.0	90	10	17	10	239.0	136.0	90	10	36	10
320	Tebuconzole	9.94	Positive	308.1	70.0	66	10	27	8	308.1	125.1	66	10	55	10
321	Tebufenozide	9.48	Positive	353.2	133.2	81	10	23	4	353.2	296.9	81	10	15	4
322	Tebufenpyrad	11.61	Positive	334.2	117.0	71	10	47	4	334.0	145.0	91	10	37	4
323	Tebuthiuron	5.78	Positive	229.1	172.1	91	10	23	20	229.1	116.2	91	10	35	8
324	Teflubenzuron	11.74	Negative	378.8	338.9	-55	-10	-18	-23	378.8	195.9	-55	-10	-34	-13
325	Tepraloxydim	8.90	Positive	342.1	250.2	101	10	21	18	342.1	166.2	101	10	25	12

No.	Compound Name	tR	Ionization	Quantitation ion MRM transition profiles						Confirmation ion MRM transition profiles					
				Precursor ion (m/z)	Product ion (m/z)	DP	EP	CE	CXP	Precursor ion (m/z)	Product ion (m/z)	DP	EP	CE	CXP
326	Terbuthylazine	8.11	Positive	230.1	174.1	81	10	23	4	230.1	104.1	86	10	41	4
327	Terbutryn	8.60	Positive	242.1	186.2	90	10	26	10	242.1	96.0	90	10	40	10
328	Tetrachlorvinphos	9.62	Positive	367.1	126.9	90	10	20	10	367.1	206.0	90	10	51	10
329	Tetraconazole	8.99	Positive	372.0	159.0	81	10	39	4	372.0	70.0	76	10	47	4
330	Thenylchlor	9.02	Positive	324.1	127.0	66	10	27	12	324.1	97.1	66	10	57	8
331	Thiabendazole	4.05	Positive	202.0	175.1	79	10	35	4	202.0	131.1	79	10	43	4
332	Thiacloprid	4.28	Positive	253.1	126.1	56	10	29	12	253.1	90.1	56	10	51	8
333	Thiamethoxam	3.61	Positive	292.0	211.0	74	10	17	4	292.0	181.0	96	10	31	4
334	Thiazopyr	9.81	Positive	397.1	377.1	151	10	31	16	397.1	335.1	151	10	41	14
335	Thidiazuron	5.50	Negative	219.0	100.0	-25	-10	-16	-9	219.0	70.9	-25	-10	-54	-11
336	Thifensulfuron-methyl	5.11	Negative	385.9	139.0	-80	-10	-28	-1	385.9	107.0	-80	-10	-74	-7
337	Thifluzamide	9.21	Positive	526.9	147.9	90	10	43	10	526.9	486.8	90	10	38	10
338	Thiobencarb	10.67	Positive	258.1	125.0	91	10	25	4	258.1	89.0	69	10	67	4
339	Thionazin	6.89	Positive	249.0	96.9	90	10	34	10	249.0	192.9	90	10	20	10
340	Tiadinil	8.72	Negative	265.9	70.9	-60	-10	-18	-11	265.9	237.8	-60	-10	-18	-13
341	Tolfenpyrad	11.72	Positive	384.2	197.3	90	10	35	10	384.2	154.2	90	10	55	10
342	Triadimefon	8.52	Positive	294.2	197.1	96	10	21	14	294.2	69.1	96	10	27	8
343	Triazophos	8.67	Positive	314.0	162.0	76	10	25	4	314.0	119.1	71	10	47	4
344	Tricyclazole	4.59	Positive	190.0	163.1	89	10	31	4	190.0	136.0	91	10	37	4
345	Trifloxystrobin	10.90	Positive	409.1	186.1	59	10	23	4	409.0	206.0	46	10	21	4
346	Triflumizole	10.99	Positive	346.0	278.0	56	10	17	4	346.0	72.9	56	10	23	4
347	Triflumuron	10.31	Positive	359.1	156.2	71	10	23	4	359.1	139.0	71	10	43	4
348	Trimethacarb	7.03	Positive	194.2	137.0	81	10	17	10	194.2	122.1	81	10	37	10
349	Triticonazole	9.06	Positive	318.1	70.0	101	10	21	8	318.1	125.1	101	10	45	10
350	Uniconazole	9.39	Positive	292.1	70.1	79	10	37	4	292.1	125.0	70	10	51	4
351	Vamidothion	3.99	Positive	288.0	145.9	56	10	17	4	288.0	118.0	56	10	31	4
352	XMC	6.29	Positive	180.1	123.0	56	10	19	14	180.1	107.0	56	10	49	8
353	Zoxamide	10.16	Positive	336.0	187.0	90	10	30	10	338.0	189.0	90	10	30	10

Table S2. Recovery results of representative pesticides that showed a difference in recovery rate depending on number of acetonitrile-hexane partition rounds (*N* = 1, 2, and 3). Each partitioning round was conducted with acetonitrile extraction using QuEChERS original salts (NaCl and $MgSO_4$) and dSPE cleanup using PSA sorbent.

Compound name	Classification	Recovery, % (RSD, %)		
		***N* = 1**	***N* = 2**	***N* = 3**
Ametryn	Triazine	60.1 (0.7)	78.7 (5.8)	80.8 (3.6)
Aspon	Organophosphate	59.8 (1.5)	81.2 (2.3)	85.7 (1.6)
Buprofezin	Others	57.4 (1.1)	78.2 (4.7)	85.4 (2.6)
Cadusafos	Organophosphate	65.2 (5.8)	81.2 (4.4)	86.0 (4.6)
Chlorpyrifos	Organophosphate	59.4 (1.6)	75.2 (4.6)	82.7 (3.5)
Diazinon	Organophosphate	66.8 (5.3)	73.9 (1.2)	87.8 (2.4)
Dimepiperate	Unclassified	55.2 (3.6)	68.0 (15.8)	72.9 (12.6)
Dimethametryn	Triazine	43.9 (1.3)	66.7 (2.6)	88.9 (5.2)
Esprocarb	Carbamate	49.5 (3.2)	64.0 (5.0)	90.1 (10.6)
Etoxazole	Unclassified	64.2 (1.6)	78.0 (2.0)	81.4 (2.3)
Fenhexamid	Others	68.2 (3.8)	79.7 (10.3)	80.7 (2.6)
Fenpropathrin	Pyrethroid	69.0 (2.0)	82.8 (8.0)	90.2 (1.5)
Fenpropimorph	Others	19.5 (1.5)	63.0 (1.2)	75.7 (2.1)
Hexythiazox	Others	62.5 (2.9)	73.7 (2.6)	74.2 (1.4)
Mepanipyrim	Others	61.0 (5.4)	72.6 (7.0)	86.5 (4.1)
Pirimiphos-ethyl	Organophosphate	58.0 (4.0)	78.7 (2.2)	82.5 (5.2)
Prometryn	Triazine	45.9 (1.8)	68.5 (3.7)	74.2 (10.9)
Propazine	Triazine	64.5 (5.5)	74.2 (4.5)	87.6 (3.1)
Pyridaben	Unclassified	60.7 (2.0)	69.7 (2.3)	73.0 (2.2)
Pyridate	Others	52.6 (2.7)	76.6 (8.2)	82.9 (4.8)
Pyrimidifen	Others	55.0 (2.0)	66.1 (2.3)	72.2 (0.2)
Spirodiclofen	Others	68.1 (1.9)	79.7 (2.1)	84.5 (0.9)
Sulprofos	Organophosphate	64.6 (2.0)	76.0 (1.7)	79.6 (1.4)
Tebufenpyrad	Others	62.2 (2.4)	80.9 (1.9)	89.0 (6.5)
Terbutryn	Triazine	50.5 (4.7)	58.5 (1.9)	78.4 (1.7)
Thiabendazole	Others	68.7 (1.5)	74.8 (7.0)	78.7 (2.9)

Table S3. Comparison of four QuEChERS extraction combinations and distribution of recovery results for 353 target pesticides. Each extraction was conducted with acetonitrile-hexane partitioning (*N* = 3) followed by dSPE cleanup using PSA.

		**No. of pesticides under detailed sample preparation conditions**			
		**QuEChERS EN 15662 salts[1]**		**QuEChERS Original salts[2]**	
**Recovery (%)**	**RSD (%)**	**MeCN[3] (12.5 mL)**	**0.1% FA[4] in MeCN (12.5 mL)**	**MeCN (12.5 mL)**	**0.1% FA in MeCN (12.5 mL)**
<10	>0	2 (0.6)	7 (2.0)	7 (2.0)	6 (1.7)
10 to 30	≤20	1 (0.3)	8 (2.3)	9 (2.5)	5 (1.4)
	>20	6 (1.7)	1 (0.3)	1 (0.3)	5 (1.4)
30 to 70	≤20	17 (4.8)	27 (7.6)	30 (8.5)	20 (5.7)
	>20	15 (4.2)	4 (1.1)	1 (0.3)	3 (0.8)
70 to 120	≤20	300 (85.0)	289 (81.9)	294 (83.3)	295 (83.6)
	>20	10 (2.8)	15 (4.2)	8 (2.3)	12 (3.4)
>120	≤20	2 (0.6)	2 (0.6)	3 (0.8)	6 (1.7)
	>20	0 (0.0)	0 (0.0)	0 (0.0)	0 (0.0)
nd[5]		0 (0.0)	0 (0.0)	0 (0.0)	1 (0.3)
Sum		353 (100)	353 (100)	353 (100)	353 (100)

[1] NaCl (1 g), $MgSO_4$ (4 g), Na_3Citrate · $2H_2O$ (1 g), Na_2HCitrate · $1.5H_2O$ (0.5 g).

[2] NaCl (1 g), $MgSO_4$ (4 g).

[3] Acetonitrile.

[4] Formic acid.

[5] Not detected.

Table S4. Recovery results of representative pesticides that showed different recovery rate depending on dSPE sorbent combination (PSA+C18, PSA, and C18). Each dSPE cleanup was conducted with acetonitrile extraction using EN 15662 salts followed by acetonitrile-hexane partitioning (*N* = 3).

Compound name	Classification	Recovery, % (RSD, %)		
		PSA+C18	PSA	C18
Azimsulfuron	Sulfonylurea	37.7 (1.9)	42.5 (17.6)	91.5 (2.8)
Bensulfuron-methyl	Sulfonylurea	39.0 (2.9)	47.3 (19.5)	89.5 (3.1)
Chlorimuron-ethyl	Sulfonylurea	28.1 (1.5)	39.5 (15.4)	79.6 (3.7)
Chlorsulfuron	Sulfonylurea	22.1 (2.2)	34.9 (12.8)	70.9 (2.4)
Clethodim	Cyclohexene oxime	67.8 (4.9)	63.2 (9.9)	82.1 (8.2)
Cyclosulfamuron	Sulfonylurea	36.1 (1.1)	49.8 (20.1)	91.8 (1.0)
Ethoxysulfuron	Sulfonylurea	22.7 (0.6)	34.7 (15.3)	83.4 (2.1)
Fenquinotrione	Triketone	4.9 (1.4)	11.0 (5.3)	54.1 (3.2)
Halosulfuron-methyl	Sulfonylurea	53.3 (8.5)	58.8 (15.3)	87.5 (9.4)
Haloxyfop	Propionic acid	3.5 (0.7)	6.4 (5.8)	91.7 (1.6)
Imazamox	Imidazolinone	2.6 (0.4)	13.4 (12.1)	70.1 (2.9)
Imazapic	Imidazolinone	3.2 (0.2)	10.6 (9.3)	70.1 (5.0)
Imazaquin	Imidazolinone	1.6 (0.2)	6.2 (6.7)	72.2 (4.0)
Imazethapyr	Imidazolinone	3.9 (0.9)	11.7 (10.7)	67.6 (3.4)
Imazosulfuron	Sulfonylurea	28.5 (1.0)	39.2 (13.9)	74.8 (2.4)
Metazosulfuron	Sulfonylurea	42.3 (4.8)	49.8 (9.7)	81.1 (2.2)
Methamidophos	Organophosphate	68.5 (5.8)	70.0 (11.3)	83.9 (3.6)
Nicosulfuron	Sulfonylurea	5.7 (0.3)	20.5 (17.3)	77.4 (4.6)
Penoxsulam	Sulfonamide	34.3 (2.8)	41.3 (17.2)	90.6 (9.7)
Probenazole	Sulfonamide	54.9 (2.2)	68.4 (14.4)	93.7 (2.9)
Pyrimisulfan	Sulfonamide	64.8 (0.9)	61.6 (5.0)	97.8 (0.4)
Saflufenacil	Sulfonamide	43.9 (8.7)	57.4 (18.7)	98.2 (2.7)
Sethoxydim A	Cyclohexene oxime	71.0 (1.9)	67.3 (1.9)	80.5 (2.0)
Spirotetramat-enol	Tetramic acid	20.8 (1.1)	36.2 (20.4)	96.5 (3.9)
Sulfentrazone	Sulfonamide	66.6 (9.5)	61.7 (14.5)	101.6 (1.1)
Tepraloxydim	Cyclohexene oxime	70.7 (18.0)	63.5 (11.0)	85.7 (4.1)
Thidiazuron	Thiadiazolylurea	68.4 (1.1)	57.3 (5.0)	94.9 (1.7)
Thifensulfuron-methyl	Sulfonylurea	16.6 (0.7)	28.7 (16.3)	84.4 (3.9)
(*cf.*) Spirotetramat	Tetramic acid	112.9 (6.4)	104.6 (21.9)	97.0 (6.2)
(*cf.*) TCMTB	Others	15.9 (3.0)	11.9 (0.3)	18.2 (5.0)

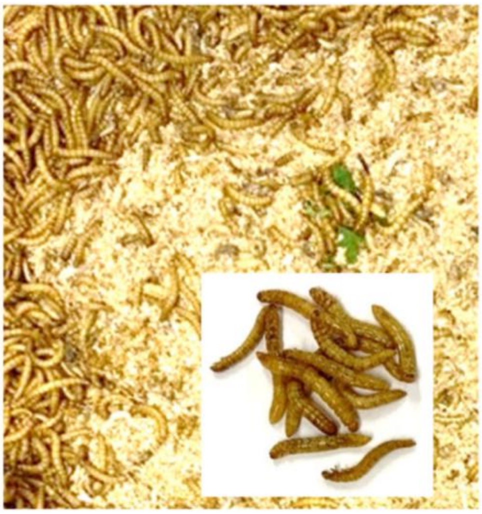

Figure S1. Mealworms grown in wheat bran.
